# The role of elevated depressive symptoms in the incident mild cognitive impairment in China: a 9-year prospective cohort study of middle-aged and older Chinese adults (2011–2020)

**DOI:** 10.3389/fpsyt.2025.1726680

**Published:** 2025-12-09

**Authors:** Shuqing Gao, Libin Xiao, Jie Lv, Jingyi Fan

**Affiliations:** Department of Psychiatry, Nanjing Youan Hospital, Nanjing, China

**Keywords:** depressive symptoms, MCI, middle-aged and older adults, CHARLS, cohort study

## Abstract

**Background:**

Although depressive symptoms are suspected to influence cognitive health, prospective evidence linking depressive symptoms to mild cognitive impairment (MCI) is scarce within large-scale Chinese cohorts. This study sought to analyze the graded association between initial depressive symptom severity and subsequent MCI risk in middle-aged and older adults in China, as well as to assess whether this relationship was moderated by other underlying factors.

**Methods:**

Using data from the China Health and Retirement Longitudinal Study (CHARLS, 2011–2020), we included 9,461 participants aged ≥45 without baseline MCI. Depressive symptoms were assessed using the CESD-10 scale and categorized as none (<10), mild–moderate (10–20), or severe (≥21). Incident MCI was identified based on standardized criteria. Data analysis was performed using Cox proportional hazards regression models, which incorporated restricted cubic splines to flexibly model potential non-linear relationships. This primary analysis was further supplemented by comprehensive subgroup and sensitivity analyses to assess the heterogeneity of treatment effects and the robustness of the findings.

**Results:**

During the nine-year follow-up period, a total of 1,276 new cases of MCI were documented. Analysis by depressive symptom severity revealed a graded increase in MCI risk relative to the asymptomatic reference group. In the fully adjusted model, participants with mild-to-moderate depressive symptoms exhibited a significantly increased risk of MCI (HR=1.363, 95%CI: 1.205–1.542, *p*<0.05), while a more pronounced risk elevation was observed in those with severe symptoms (HR=1.468, 95%CI: 1.123–1.918, *p*<0.05). Furthermore, each 1-SD (5.83 points) increase in CES-D score was associated with a 2.90% higher risk of MCI (HR=1.029, 95% CI: 1.020-1.039, *p*<0.001). Restricted cubic splines confirmed a significant linear dose-response relationship (*p* for nonlinear=0.419). Education significantly moderated the association, with a protective effect observed in individuals with higher education. The findings remained consistent when assessed through various sensitivity analyses.

**Conclusion:**

Baseline depressive symptoms are independently and dose-dependently associated with higher MCI risk in Chinese adults aged 45 and above. Education serves as a significant moderator. Integrating depression screening into MCI prevention strategies is recommended, particularly in less-educated populations.

## Introduction

Global population aging represents a critical demographic transition with profound implications for public health and societal systems worldwide (1). This shift has significantly increased the global burden of age-related chronic conditions, leading to higher risks of functional disability and care dependency (2). Among these, dementia represents a particularly severe and fast-growing public health crisis. Marked by irreversible cognitive decline, dementia is the fastest-growing major neurological disorder globally (3, 4). By 2050, the number of people living with dementia is projected to nearly triple, with population aging accounting for the majority (92%) of this rise (5, 6). The condition’s gradual progression and earlier potential manifestation heighten the importance of preemptive measures, including screening and early treatment strategies (7). China provides a particularly compelling context for studying age-related cognitive disorders, as it is home to the world’s largest aging population and is projected to become a super-aged society by 2065 (1, 8). Accounting for over 25% of global dementia burden, China reported 13.1 million cases within a worldwide total of 51.6 million in 2019 (9). Findings from this context are therefore of broad relevance to other nations experiencing similar demographic transitions.

Mild cognitive impairment (MCI) presents a considerable global health burden, estimated at 15.56% in populations aged 50 and older (10), with studies in rural China reporting rates exceeding 25% among the elderly (11). As MCI constitutes a preclinical stage of dementia (12), its high prevalence underscores its importance as a crucial target for preventive interventions. MCI progresses to dementia at an annual rate of 39.2% (13), highlighting the urgency of early detection and management. While epidemiological studies have identified nine risk factors for MCI (14), their underlying neurobiological mechanisms remain inadequately elucidated.

Recent research efforts have increasingly turned their attention to understanding the complex, often bidirectional, relationship linking depression and cognitive dysfunction. Accumulating research supports the classification of depression as a major independent predictor of dementia, with a dose-response relationship observed (15, 16). Notably, late-life depressive symptoms may often represent a prodromal stage of dementia (17), suggesting depressive symptoms may actively contribute to neurodegeneration.

Despite considerable research, the exact role of depression in the development of MCI is still unclear and methodologically contested, representing a critical unanswered question in the field. Inconsistencies across existing studies complicate definitive conclusions. For instance, one investigation emphasized that anxiety, rather than depression, interacted with β-amyloid (Aβ) deposition to hasten progression from normal cognition to MCI (18). In contrast, several longitudinal studies—including a 4-year Chinese cohort analysis and a 10-year European follow-up—have identified depressive symptoms as an independent predictor of MCI, noting a significant graded dose-response relationship (19, 20). These inconsistencies could arise from differences in sample characteristics, study design, measurement methods, or statistical techniques, underscoring the importance of rigorously conducted prospective studies with sufficient scale and duration.

To bridge this methodological and epidemiological gap, particularly the lack of a formal assessment of the non-linear relationship between depressive symptoms and MCI risk and a rigorous examination of educational attainment as a potential effect modifier, we established a prospective cohort drawing upon 9-year nationally representative longitudinal data (2011–2020) from Chinese adults aged 45 years and older. We hypothesize that baseline depressive symptoms predict a higher risk of incident MCI a dose-response fashion, and to examine the potential moderating effects of underlying factors on this association. This research aims to produce high-grade epidemiological data to underpin evidence-based early preventive measures, with its findings intended to contribute to global healthy aging initiatives and implications for aging populations worldwide facing similar cognitive health challenges.

## Methods

### Study design and population

Data for this secondary analysis was sourced from the China Health and Retirement Longitudinal Study (CHARLS). The CHARLS database incorporates five waves of data collection, carried out in 2011 (baseline), 2013, 2015, 2018, and 2020. The employed sampling framework successfully enrolled a total of 17,708 participants, encompassing approximately 10,000 households in this nationally representative survey (21). The fully anonymized dataset used in this study is accessible to the public through the official CHARLS website (https://charls.pku.edu.cn/).

**Figure 1:**
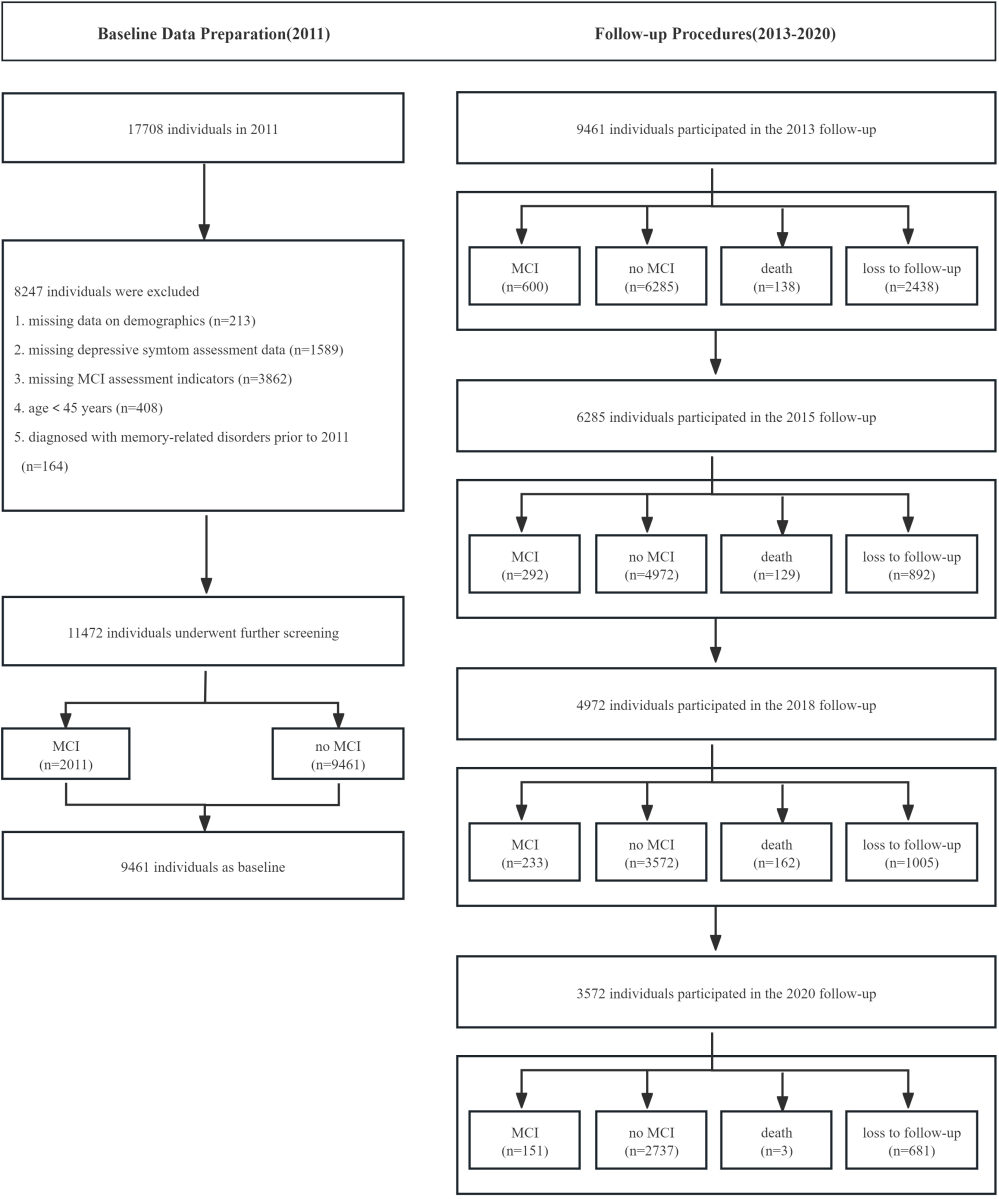
Flowchart of participant selection from the CHARLS cohort (2011–2020). This flowchart illustrates the sequential exclusion process applied to the baseline (2011) sample (n=17,708) to derive the final analytical cohort free of baseline MCI (n=9,461), followed by the tracking of incident MCI cases and attrition at each follow-up wave (2013, 2015, 2018, 2020). CHARLS, China Health and Retirement Longitudinal Study; MCI, mild cognitive impairment.

This study employed longitudinal data from the nationwide CHARLS cohort, with follow-up assessments spanning 2011 to 2020. Through sequential exclusions, we established our analytical cohort: (1) 213 excluded for incomplete basic information; (2) 1,589 excluded for missing depression indicators; (3) 3,862 excluded for missing cognitive assessments; (4) 408 excluded for age<45 years; (5) 164 excluded for pre-baseline diagnosis of memory-related disorders; (6) 2,011 excluded for meeting MCI diagnostic criteria at baseline. Finally, 9,461 adults aged ≥ 45 years with complete depression/cognitive data and without baseline MCI were enrolled for prospective follow-up (2013, 2015, 2018, 2020 waves). Throughout the follow-up period (2013–2020), 1,276 incident MCI cases were identified within the baseline MCI-free cohort (**Figure 1**). Ethical approval for this study was obtained from the Biomedical Ethics Committee of Peking University (IRB00001052-11015). All participants provided written informed consent. This analysis adhered to STROBE guidelines (22).

### Data assessment and definitions

#### Assessment of exposure (depressive symptoms)

Depressive symptomatology was evaluated utilizing the 10-item Center for Epidemiologic Studies Depression Scale (CESD-10), which produces a total score between 0 and 30. Scores on this scale correspond to the self-reported severity and persistence of depressive symptoms experienced during the previous week. Respondents rated the occurrence of each symptom using a 4-point Likert-type option, ranging from 0 (“rarely or none of the time” [<1 day]) to 3 (“most or all of the time” [5–7 days]). Scale items encompassed: being bothered by minor issues; difficulty concentrating; depressed mood; feeling that everything required effort; hopefulness (reverse-scored); fearfulness; restless sleep; happiness (reverse-scored); loneliness; and lack of motivation. Positively worded items (hopefulness and happiness) were reverse-scored prior to summation. The scale has held good validity for use among adult populations in China (23). Psychometric evaluations demonstrated strong reliability and validity: baseline Cronbach’s α=0.805, KMO=0.880, Bartlett’s test P<0.001 (24). To assess associations with MCI, participants were categorized using two complementary grouping strategies based on CESD-10 scores. Participants were categorized according to established grouping protocols from relevant literature. The first strategy employed a clinically relevant dichotomization wherein participants scoring below the established cutoff of 10 were categorized as having no elevated depressive symptoms, while those scoring ≥ 10 were classified into having elevated depressive symptoms, reflecting a clinically significant symptom burden associated with increased depression risk (25). The second strategy stratified participants by symptom severity into three distinct categories: no elevated symptoms (scores<10), mild-moderate symptoms (scores 10–20), and severe symptoms (scores ≥ 21) (26, 27). The threshold of 21 was selected based on its established association with high depression risk, while the 20-point demarcation aligned with common clinical severity gradations. All categorizations reflected self-reported symptom frequency rather than clinical diagnoses, with higher categories indicating progressively greater likelihood of clinical depression.

#### Assessment of outcomes (MCI)

Cognitive function in the CHARLS was assessed using a measurement framework aligned with that of the U.S. Health and Retirement Study (HRS) to facilitate cross-national comparability. Evaluations included two principal components: the Word Recall Test (WRT) and the Mental Status Test (MST) (28). The WRT consisted of two phases: immediate recall and delayed recall. Participants were read a list of ten common words and asked to recall as many as possible immediately (scored 0–10) and again after a delay during which other tasks were completed (also scored 0–10). Scores for the Word Recall Test (WRT) were calculated by summing one point per correct response, yielding a cumulative score that could vary from 0 to 20. The MST incorporated the 10-item Telephone Interview for Cognitive Status (TICS-10) and a Figure drawing task. The TICS-10 evaluated two domains: computational ability and orientation. For computational ability, participants performed five consecutive serial subtractions of 7 from 100, earning 1 point for each correct subtraction (scored 0-5). Orientation was determined by evaluating the participant’s knowledge of the current year, month, date, day of the week, and season. Each correct response contributed one point, yielding a maximum score of 5. Subsequently, the drawing task required the replication of a diagram featuring two intersecting pentagons. A single point was allotted for a correct rendition. The aggregate score of these two tasks, therefore, spanned from 0 to 11.

A composite metric of overall cognition was derived by aggregating scores across four distinct domains: memory (0-20), computation (0-5), orientation (0-5), and drawing (0-1), with a theoretical maximum of 31 points (29, 30). Currently, there is no gold standard for defining MCI. In line with prior studies (31, 32), the classification of MCI was based on the Aging-Associated Cognitive Decline (AACD) framework. Under this approach, participants were classified as having MCI based on a composite cognitive score falling one or more standard deviations below demographically adjusted normative values. Age stratification was performed using five-year intervals, and the AACD cutoffs were applied within each age group to identify MCI cases.

#### Assessment of covariates

The selection of covariates was based on established and potential risk factors for MCI, as well as prior studies utilizing the CHARLS dataset, to ensure comparability and theoretical relevance (29, 33, 34) (Wave 1, 2011): (a) Sociodemographic profile: sex, age, educational level, marital status, and household registration (hukou); (b) Anthropometric assessments: body mass index (BMI), height, body weight, waist circumference, systolic blood pressure (SBP), and diastolic blood pressure (DBP); (c) Lifestyle behaviors: smoking status, alcohol consumption, and nightly sleep duration; (d) Clinical comorbidities: dyslipidemia, hypertension, diabetes, kidney disease, liver disease, cardiovascular disease, and stroke; (e) Medication use: lipid-lowering agents, antihypertensive drugs, hypoglycemic medications, cardioprotective drugs, and cerebroprotective agents; (f) Laboratory assays: glycated hemoglobin (HbA1c), fasting blood glucose (FBG), triglycerides (TG), total cholesterol (TC), high-density lipoprotein cholesterol (HDL-c), low-density lipoprotein cholesterol (LDL-c), blood urea nitrogen (BUN), uric acid (UA), and C-reactive protein (CRP).

### Management of missing data

This study applied distinct missing data handling strategies based on variable characteristics. The core independent variable (CESD-10 scores) and key covariates (age, sex, education, marital status, residence) contained no missing values and required no imputation. To handle covariates with missing data, we employed the Multiple Imputation by Chained Equations (MICE) approach under a Markov Chain Monte Carlo framework, running the algorithm across 50 iterations to yield five imputed datasets. Regarding the dependent variable (MCI status), subjects lost to follow-up (including deaths) were retained in the analytical cohort without imputation but treated as right-censored observations to maximize statistical power while preserving data authenticity. Mortality cases were specifically flagged for subsequent sensitivity analyses employing competing risk methodologies.

### Statistical analysis

The study cohort (n=9,461) was stratified into three categories according to CESD-10 scores: no elevated depressive symptoms (< 10), mild to moderate symptoms (10–20), and severe symptoms (≥ 21). Continuous variables are presented as mean (standard deviation), with group comparisons performed using one-way analysis of variance (ANOVA). Categorical variables were summarized as counts and proportions, and group differences were evaluated by means of the chi-square test.

To evaluate the association between depressive symptoms and the subsequent development of MCI in a prospective cohort of individuals aged ≥45, we utilized Cox proportional hazards regression. Analyses were conducted across a sequence of nested models with increasing degrees of adjustment: a baseline without adjustment for covariates (Model 1); demographically adjusted (Model 2); further adjusted for anthropometric and lifestyle variables (Model 3); additionally incorporating medical history (Model 4); and finally, further adjusted for laboratory measures (Model 5). To preclude concerns of multicollinearity, this study calculated variance inflation factors (VIFs) for all covariates prior to modeling. With all values falling below 5, the results are presented as hazard ratios (HRs) with associated 95% confidence intervals (CIs), confirming that collinearity did not substantially influence the model estimates.

Furthermore, using restricted cubic splines (RCS) within a multivariable Cox model, we examined the dose-response relationship linking CESD-10 scores to MCI incidence. The application of RCS at the 5th, 35th, 65th, and 95th percentiles allowed for a flexible examination of potential nonlinear associations. Additionally, potential effect modification was assessed through subgroup analyses, including stratification by age (< 60 years, ≥ 60 years), sex, household registration status, educational attainment, marital, smoking status, alcohol consumption, presence of hypertension, presence of dyslipidemia, presence of diabetes, history of cardiovascular disease, and history of stroke. Interaction analyses were performed within these subgroups.

To assess the robustness of the depressive symptoms–MCI relationship, we performed two sensitivity analyses. First, to address potential temporal variations in assessment tools or diagnostic criteria, a stratified Cox model was fitted using assessment year (2013, 2015, 2018, 2020) as a stratification variable, allowing separate baseline hazards per period and mitigating bias from time-dependent confounders. The exposure and covariates remained consistent with Model 5 of the primary analysis. Second, to address the competing risk of death and utilize all available intermittent follow-up data, a multinomial logistic regression model was implemented using a composite outcome variable (0: MCI-free; 1: incident MCI; 2: all-cause death), including all available observations except those lost to follow-up. This model estimated associations of depressive symptoms with both MCI and death, using the MCI-free group as reference, with covariate adjustment matching the primary model. All data preprocessing and statistical analyses were performed using R 4.1.0, SPSS 23.0 and Graphpad Prism 9.2. Statistical significance was defined as a two-tailed P-value of less than 0.05.

## Results

### Baseline characteristics of participants

Of the 17,708 individuals initially enrolled in the 2011 survey wave, 9,461 met the inclusion criteria following the application of sequential exclusion steps and comprised the final analytical cohort. **Figure 1** depicts the detailed study flowchart. The baseline sociodemographic and clinical characteristics of the participants are summarized in **Table 1**, grouped by three levels of symptom severity as assessed at the 2011 survey: no elevated symptoms (CESD-10 scores<10, n=6,636), mild-moderate symptoms (scores 10-20, n=2,494), and severe symptoms (scores ≥ 21, n=331). The overall mean CESD-10 score at baseline was 7.33 (5.83). Compared to the no elevated symptoms group, participants with worsening depressive symptoms (the combined mild-moderate and severe symptom groups) was characterized by: advanced age, a greater representation of females, higher proportion of rural residents, and lower proportion with college education; lower measurements of waist circumference, weight, DBP, and UA; higher levels of HDL-c and HbA1c; and significantly shorter nighttime sleep duration. The prevalence rates of hypertension, diabetes, cardiovascular disease, and stroke were significantly elevated (*p*<0.05). Notably, cognition scores progressively declined with increasing severity of depressive symptoms (*p* < 0.001). Demographic and clinical profiles of participants at enrollment, stratified by binary depressive symptom classification and incident MCI status during follow-up, are summarized in **Supplementary Tables S1** and **S2**. Additionally, the distribution and extent of missing data across baseline characteristics are summarized in **Supplementary Table S3**. A comparison of the distribution of variables before multiple imputation is presented in **Supplementary Table S4**.

**Table 1:** Baseline characteristics of participants overall and by severity of depressive symptoms.

Characteristics	Overall	Symptom severity stratification			*P*-value
		No elevated symptoms	Mild-moderate symptoms	Severe symptoms	
N	9461	6636	2494	331	
Age, years	58.16 (9.02)	57.80 (9.00)	58.89 (9.04)	59.80 (8.68)	<0.001
Gender					<0.001
Male	5203 (55.0)	3878 (58.4)	1175 (47.1)	150 (45.3)	
Female	4258 (45.0)	2758 (41.6)	1319 (52.9)	181 (54.7)	
Marital status					<0.001
Unmarried	1354 (14.3)	795 (12.0)	484 (19.4)	75 (22.7)	
Married	8107 (85.7)	5841 (88.0)	2010 (80.6)	256 (77.3)	
Education level					<0.001
Elementary school or below	5211 (55.1)	3363 (50.7)	1609 (64.5)	239 (72.2)	
Middle school	3626 (38.3)	2732 (41.2)	806 (32.3)	88 (26.6)	
College or above	624 (6.6)	541 (8.2)	79 (3.2)	4 (1.2)	
Residence					<0.001
Urban	2763 (29.2)	2172 (32.7)	549 (22.0)	42 (12.7)	
Rural	6698 (70.8)	4464 (67.3)	1945 (78.0)	289 (87.3)	
BMI, kg/m2	24.20 (26.62)	24.57 (31.68)	23.33 (3.80)	23.31 (3.69)	0.114
Waist	85.15 (12.55)	85.58 (12.61)	84.34 (12.03)	82.77 (14.41)	<0.001
Weight	61.09 (11.88)	62.22 (11.92)	58.45 (11.21)	58.10 (12.37)	<0.001
Height	1.60 (0.09)	1.61 (0.09)	1.58 (0.08)	1.58 (0.08)	<0.001
SBP, mmHg	129.26 (20.80)	129.50 (20.58)	128.61 (21.12)	129.52 (22.72)	0.183
DBP, mmHg	75.92 (12.18)	76.26 (12.15)	75.09 (12.11)	75.33 (12.97)	<0.001
Smoking status					0.010
Current	3194 (33.8)	2282 (34.4)	798 (32.0)	114 (34.4)	
Former	977 (10.3)	717 (10.8)	231 (9.3)	29 (8.8)	
Never	5290 (55.9)	3637 (54.8)	1465 (58.7)	188 (56.8)	
Drinking status					<0.001
Current	3233 (34.2)	2431 (36.6)	720 (28.9)	82 (24.8)	
Former	806 (8.5)	508 (7.7)	251 (10.1)	47 (14.2)	
Never	5422 (57.3)	3697 (55.7)	1523 (61.1)	202 (61.0)	
Nighttime sleep duration	33.25 (42.21)	6.72 (1.56)	5.93 (1.91)	5.24 (1.96)	<0.001
Dyslipidemia					0.177
No	8374 (88.5)	5900 (88.9)	2184 (87.6)	290 (87.6)	
Yes	1087 (11.5)	736 (11.1)	310 (12.4)	41 (12.4)	
Hypertension					<0.001
No	6961 (73.6)	4965 (74.8)	1776 (71.2)	220 (66.5)	
Yes	2500 (26.4)	1671 (25.2)	718 (28.8)	111 (33.5)	
Diabetes					0.030
No	8839 (93.4)	6228 (93.9)	2308 (92.5)	303 (91.5)	
Yes	622 (6.6)	408 (6.1)	186 (7.5)	28 (8.5)	
Kidney disease					<0.001
No	8920 (94.3)	6338 (95.5)	2301 (92.3)	281 (84.9)	
Yes	541 (5.7)	298 (4.5)	193 (7.7)	50 (15.1)	
Liver disease					<0.001
No	9110 (96.3)	6419 (96.7)	2385 (95.6)	306 (92.4)	
Yes	351 (3.7)	217 (3.3)	109 (4.4)	25 (7.6)	
Heart disease					<0.001
No	8242 (87.1)	5902 (88.9)	2081 (83.4)	259 (78.2)	
Yes	1219 (12.9)	734 (11.1)	413 (16.6)	72 (21.8)	
Stroke					<0.001
No	9252 (97.8)	6530 (98.4)	2410 (96.6)	312 (94.3)	
Yes	209 (2.2)	106 (1.6)	84 (3.4)	19 (5.7)	
Lipid-lowering treatment					0.017
No	8941 (94.5)	6300 (94.9)	2333 (93.5)	308 (93.1)	
Yes	520 (5.5)	336 (5.1)	161 (6.5)	23 (6.9)	
Antihypertensive treatment					<0.001
No	7582 (80.1)	5389 (81.2)	1939 (77.7)	254 (76.7)	
Yes	1879 (19.9)	1247 (18.8)	555 (22.3)	77 (23.3)	
Hypoglycemic treatment					0.025
No	9036 (95.5)	6363 (95.9)	2360 (94.6)	313 (94.6)	
Yes	425 (4.5)	273 (4.1)	134 (5.4)	18 (5.4)	
Cardioprotective therapy					<0.001
No	8704 (92.0)	6215 (93.7)	2214 (88.8)	275 (83.1)	
Yes	757 (8.0)	421 (6.3)	280 (11.2)	56 (16.9)	
Cerebroprotective therapy					<0.001
No	9349 (98.8)	6580 (99.2)	2449 (98.2)	320 (96.7)	
Yes	112 (1.2)	56 (0.8)	45 (1.8)	11 (3.3)	
HbA1c, %	5.25 (0.82)	5.24 (0.79)	5.29 (0.87)	5.31 (1.03)	0.005
FBG, mg/dL	110.40 (37.93)	110.02 (36.07)	111.55 (42.46)	109.38 (38.10)	0.203
TG, mg/dL	136.86 (101.76)	138.56 (105.00)	132.86 (91.36)	133.01 (109.12)	0.046
TC, mg/dL	192.69 (38.60)	192.25 (38.53)	194.05 (38.88)	191.38 (37.72)	0.115
HDL-c, mg/dL	49.85 (15.13)	49.43 (15.08)	50.92 (15.30)	50.06 (14.57)	<0.001
LDL-c, mg/dL	116.24 (35.68)	115.76 (35.80)	117.65 (35.60)	115.25(33.85)	0.069
BUN, mg/dL	15.62 (4.39)	15.68 (4.40)	15.52 (4.38)	15.09 (4.32)	0.023
UA, mg/dL	4.59 (1.28)	4.66 (1.29)	4.44 (1.25)	4.24 (1.14)	<0.001
CRP, mg/dL	2.80 (7.77)	2.71 (7.60)	3.04 (8.26)	2.85 (7.34)	0.190
CESD score	7.33 (5.83)	4.19 (2.77)	13.58 (2.96)	23.31 (2.21)	<0.001
Cognition score	17.52 (3.67)	18.00 (3.68)	16.49 (3.38)	15.64 (3.52)	<0.001

Data are presented as mean (standard deviation) for continuous variables and n (%) for categorical variables. P-values were derived from one-way ANOVA for continuous variables and the chi-square test for categorical variables across the three depressive symptom groups.

BMI, body mass index; SBP, systolic blood pressure; DBP, diastolic blood pressure; HbA1c, glycosylated hemoglobin; FBG, fasting blood glucose; TG, triglycerides; TC, total cholesterol; HDL-c, high-density lipoprotein cholesterol; LDL-c, low-density lipoprotein cholesterol; BUN, blood urea nitrogen; UA, uric acid; CRP, C-reactive protein.

### Association of depressive symptoms with MCI

**Figure 2:**
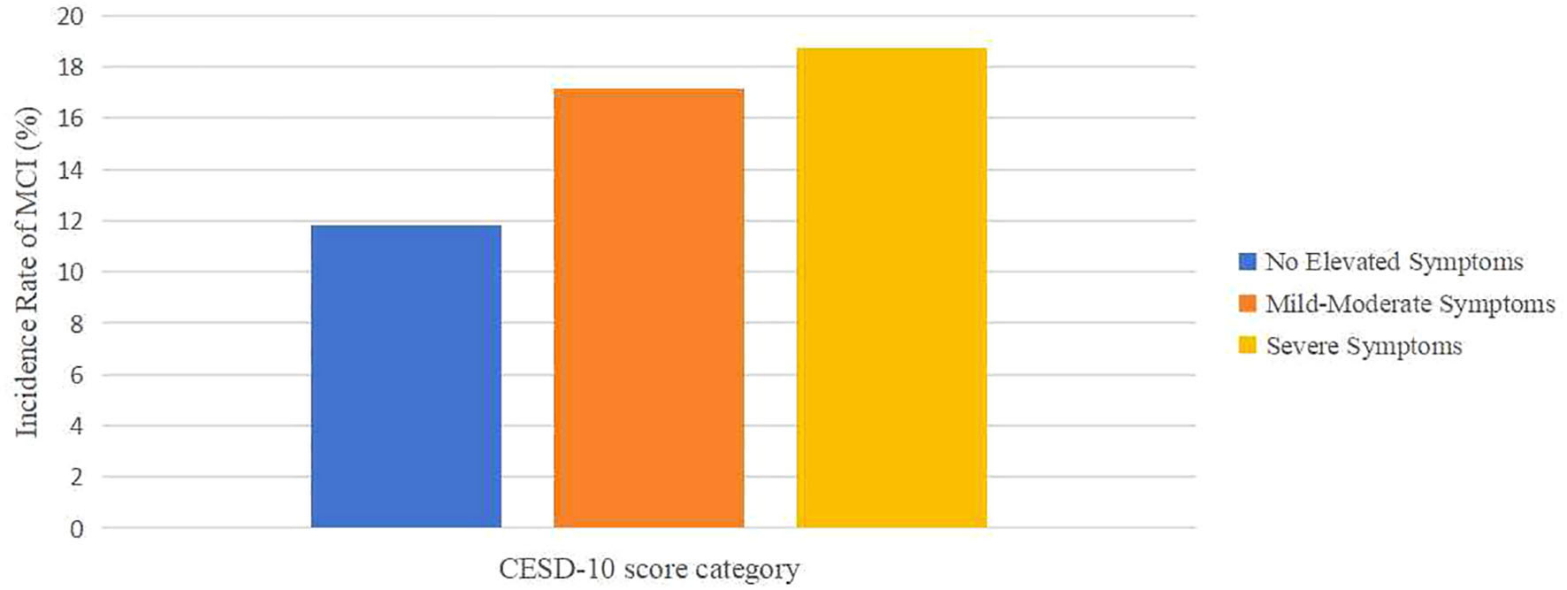
Incidence rate of new-onset MCI cases according to the severity of depressive symptoms. CESD-10, 10-item Center for Epidemiologic Studies Depression Scale.

**Figure 3:**
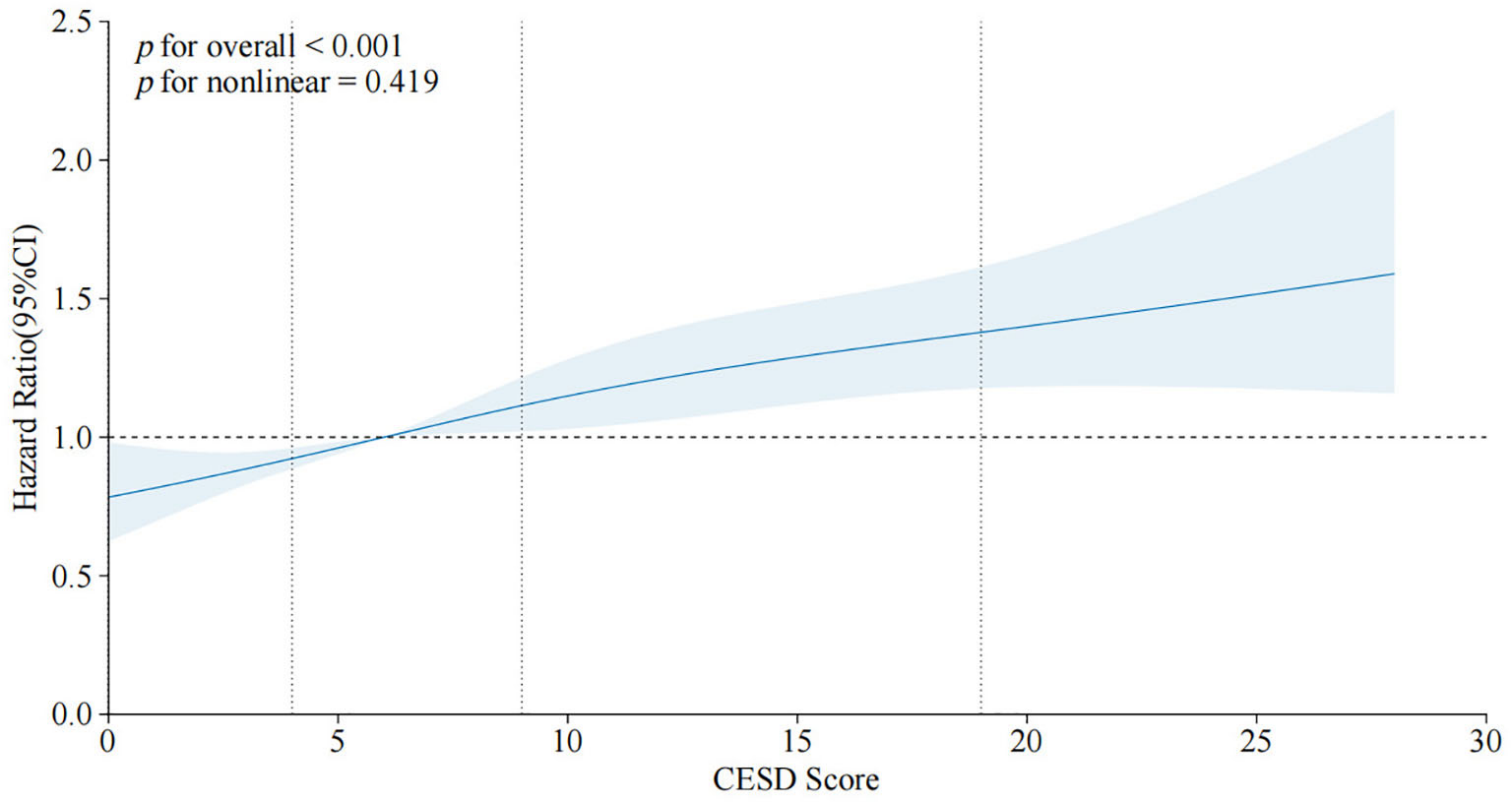
Dose-response relationship between CESD-10 scores and risk of incident MCI. The solid line represents the hazard ratio (HR), and the shaded area represents the 95% confidence interval (95% CI), derived from a multivariable-adjusted restricted cubic spline Cox regression model with 4 knots at the 5th, 35th, 65th, and 95th percentiles of the CESD-10 score distribution. The reference value (HR=1) was set at a CESD-10 score of 0. The model was adjusted for demographics, anthropometric measurements, lifestyle factors, medical history, and laboratory examinations (Model 5). The P-value for overall association was <0.001, and for nonlinearity was 0.419. HR, hazard ratio; CI, confidence interval.

Over the follow-up period spanning from 2011 to 2020, with a median duration of 9 years, 1,276 incident MCI cases (13.5%) were identified among 9,461 baseline MCI-free participants in the CHARLS cohort. As shown in **Figure 2**, the numbers of new-onset MCI cases in the three groups were 786 (11.84%), 428 (17.16%), and 62 (18.73%) respectively. The incidence rates of MCI increased progressively with the severity of baseline depressive symptoms, (ID=20.29, 95% CI: 18.90-21.76) per 1,000 person-years in the group with no elevated symptoms, (ID=32.28, 95% CI: 29.29-35.48) in the mild-moderate symptoms group, and (ID=39.77, 95% CI: 30.49-50.98) in the severe symptoms group. Multivariable Cox models revealed a significant monotonic dose-response relationship, wherein increasing severity of depressive symptoms was associated with a progressively higher risk of incident MCI (*p* for trend <0.001), with detailed distribution presented in **Table 2**. Compared to those without depressive symptoms, mild-moderate symptomatic participants showed a significant risk elevation of 36.3% for subsequent MCI development (HR=1.363, 95%CI: 1.205–1.542, *p <*0.05), while severe symptoms elevated risk by 46.8% (HR=1.468, 95%CI: 1.123–1.918, *p <*0.05). Per 1-SD (5.83 points) increase in CESD score, MCI risk increased by 2.90% (HR=1.029, 95% CI: 1.020-1.039, *p <*0.01). Restricted cubic splines confirmed a positive linear association (overall *p <*0.001) without significant nonlinearity (*p* for nonlinear=0.419): as shown in **Figure 3**, the hazard ratio increased near-linearly when CESD >10. To ensure the robustness of the Cox regression model, multicollinearity diagnostics were performed for all included variables; all VIF estimates are provided in **Supplementary Table S5**.

**Table 2:** Association between the severity of depressive symptoms and MCI incidence.

Characteristics	Symptom severity stratification			*P* for trend	Continuous
	No elevated symptoms	Mild-moderate symptoms	Severe symptoms		Per 1 SD increase
No. of new-onset MCI/totals	786/6636	428/2494	62/331	–	–
Case, n(%)	786 (11.84)	428 (17.16)	62 (18.73)	–	–
ID (95%CI) per 1,000 person-years	20.29 (18.90-21.76)	32.28 (29.29-35.48)	39.77 (30.49-50.98)	–	–
Model 1, HR(95% CI)	Ref	1.594 (1.417-1.794)**	1.950 (1.506-2.526)**	<0.001	1.045 (1.036-1.054)**
Model 2, HR(95% CI)	Ref	1.357 (1.205-1.529)**	1.462 (1.127-1.897)**	<0.001	1.028 (1.019-1.037)**
Model 3, HR(95% CI)	Ref	1.346 (1.192-1.520)**	1.451 (1.114-1.890)*	<0.001	1.028 (1.018-1.037)**
Model 4, HR(95% CI)	Ref	1.364 (1.206-1.542)**	1.455 (1.114-1.901)*	<0.001	1.029 (1.020-1.039)**
Model 5, HR(95% CI)	Ref	1.363 (1.205-1.542)*	1.468 (1.123-1.918)*	<0.001	1.029 (1.020-1.039)**

Model 1, unadjusted; Model 2, adjusted for demographic characteristics (age, sex, education, marital status, residence)**;** Model 3, Model 2 + anthropometric measurements (BMI, waist) and lifestyle factors (smoking, drinking, nighttime sleep duration)**;** Model 4, Model 3 + medical history (hypertension, dyslipidemia, diabetes, heart disease, stroke, kidney disease, liver disease)**;** Model 5, Model 4 + laboratory examinations (HbA1c, HDL-c, LDL-c, TG, TC, UA, CRP, BUN).

HR, hazard ratio; CI, confidence interval; SD, standard deviation; Ref, reference group; ** *p*<0.001; * *p*<0.05.

### Subgroup analysis

**Figure 4:**
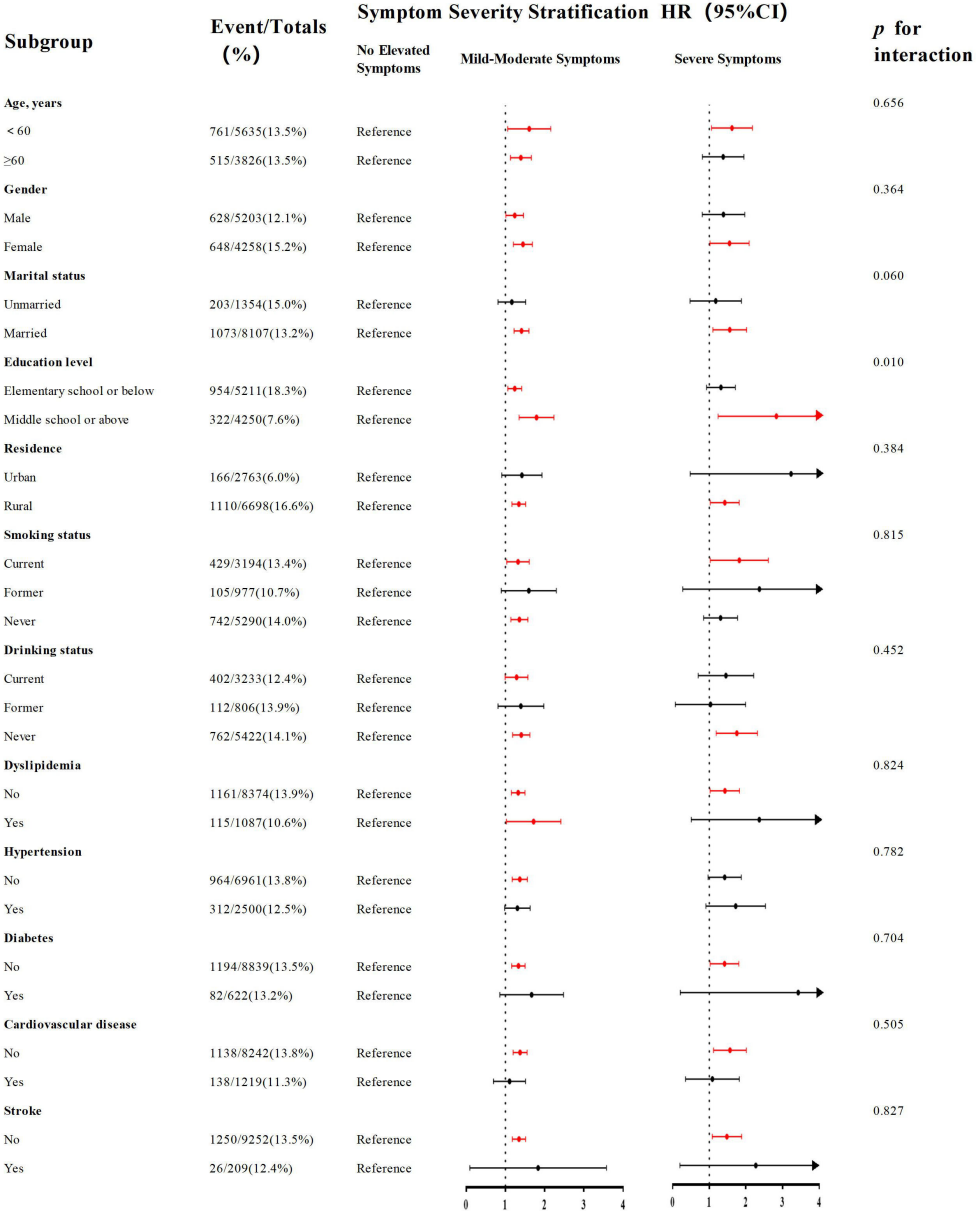
Subgroup analyses of the association between the severity of depressive symptoms and risk of incident MCI. Forest plot displays hazard ratios (squares) and 95% confidence intervals (horizontal lines) for the association between mild-moderate depressive symptoms (vs. no symptoms) and MCI risk across various subgroups. The size of the square represents the sample size of the subgroup. The model was fully adjusted (Model 5). P for interaction was calculated.

As shown in **Figure 4**, subgroup analyses identified a statistically significant moderating role of educational attainment in the relationship linking depressive symptoms to MCI risk (*p* for interaction=0.010). Among individuals with elementary school or below (n=5,211, events=954), the hazard of MCI was significantly greater in individuals with mild-moderate symptoms than in their counterparts without elevated symptoms (HR=1.230, 95% CI: 1.067-1.417, *p* =0.004). However, although an elevated risk of MCI was observed among participants with severe depressive symptoms, this association was not significant (HR=1.279, 95% CI: 0.946-1.729, *p*=0.110). Conversely, educational attainment of middle school or above (n=4,250; events=322) was associated with a more pronounced and graded dose-response relationship. Participants with mild-moderate symptoms exhibited a markedly elevated risk of developing MCI relative to the reference group (HR=1.757, 95% CI: 1.370-2.253, *p*<0.001), and the risk was even more pronounced for those with severe depressive symptoms (HR=2.535, 95% CI: 1.412-4.551, *p*=0.002). Details can be seen in **Supplementary Table S6**.

### Sensitivity analysis

When accounting for potential temporal variations in assessment protocols across follow-up waves by stratifying on survey year, the association remained significant for mild-moderate depressive symptoms (HR=1.201, 95% CI: 1.062-1.358, *p*<0.05) but was attenuated for severe symptoms (HR=1.167, 95% CI: 0.891-1.528, *p*=0.261). To address competing risk from death, we employed a multinomial model. The findings regarding mild to moderate symptomatic presentations aligned with those of the primary analysis (OR=1.306, 95% CI: 1.139-1.498, *p*<0.001). The odds ratio for severe symptoms pointed towards an increased risk, albeit with borderline significance (OR=1.343, 95% CI: 0.992-1.819, *p*=0.056). **Supplementary Table S7** provides a comprehensive breakdown of these results.

## Discussion

Drawing upon a decade of longitudinal follow-up (2011–2020) within the CHARLS cohort, this investigation revealed that baseline depressive symptoms constitute an independent predictor for the onset of MCI in middle-aged and older adults in China. A dose-response gradient was observed, whereby greater baseline depressive symptom severity corresponded to elevated subsequent MCI risk. Importantly, educational attainment emerged as a significant effect modifier, with higher levels of education attenuating the positive relationship between depressive symptoms and MCI incidence, suggesting education may function as a protective cognitive resource. These findings provided long-term epidemiological evidence from a Chinese population supporting depressive symptoms as a potential modifiable risk factor for MCI.

While derived from the Chinese context, our findings are empirically consistent with prior studies, thereby enabling them to resonate with and make significant contributions to a pressing global health challenge. Based on a 10-year follow-up period utilizing data from the European SHARE database, Han et al. also documented that depressive symptoms were a significant predictor for elevated MCI risk, with notably higher risk observed in women and older adult subgroups (20). Consistent with this finding, a longitudinal analysis by Zhou et al., conducted among elderly residents of rural China, found that deteriorating depressive symptoms were significantly associated with faster cognitive decline and a greater likelihood of developing MCI (35). Furthermore, employing latent class analysis, Du et al. revealed that cognitive status among older adults in China follows a dynamically evolving trajectory, demonstrated a close association between depression and transitions in cognitive states, and identified education as a significant moderating factor—results that are strongly congruent in agreement with the principal findings of the present analysis (36). In conclusion, studies spanning from Europe to China and across urban-rural divides have yielded convergent evidence. This body of work identifies depressive symptoms as a robust and potentially universal risk factor for MCI, with education emerging as a factor conferring a protective effect.

Notably, depressive symptoms could have divergent effects on cognitive function may differ according to the stage of cognitive impairment. Lee et al. observed a more pronounced correlation between depressive symptoms and cognitive deficits in individuals with MCI compared to those with AD, especially within the domains of executive function and memory (37). This finding implies that depressive symptoms may exert a stronger adverse effect on cognition during the early stages of cognitive decline, such as MCI, thereby reinforcing the rationale for regarding depression as a significant risk factor for MCI. Alternatively, socio-structural factors may also shape the relationship linking depressive symptoms to MCI. Using nationally representative data from the U.S. HRS-HCAP, Manly et al. documented substantial disparities in MCI prevalence across racial and educational strata, with a higher burden of disease among socioeconomically disadvantaged and minority populations (38). This aligns with the moderating role of education identified in our analysis and underscores the importance of integrating health equity perspectives into strategies for preventing and managing depression and cognitive impairment.

Despite growing recognition of the association of depressive symptoms with MCI, substantial gaps persist in the screening and diagnosis of MCI within primary care and community-based settings. Research by Kulshreshtha et al. showed that among older adults receiving primary care, up to 62.3% of MCI cases went unrecognized, and 12.3% of dementia cases were undiagnosed, with cognitive impairment risk being particularly pronounced among African Americans (39). This result indicates a notable “diagnostic gap” for MCI. Furthermore, traditional cognitive screening tools such as the MMSE have limitations in sensitivity and cultural fairness. Jannati et al. pointed out that the MMSE had poor ability to detect early cognitive impairment and was susceptible to educational and cultural backgrounds, whereas emerging digital cognitive assessment tools (such as DCR) offer higher sensitivity, less cultural bias, and were easier to administer (40). Therefore, while targeting depressive symptoms as an intervention point for MCI, it is also necessary to promote more efficient, equitable, and easily implementable cognitive assessment tools to improve early detection rates.

Regarding pathophysiological mechanisms, depressive symptoms and MCI may share some neurobiological underpinnings. Neuroimaging studies have shown that both exhibit common gray matter atrophy in multiple neural substrates (e.g., the hippocampus, amygdala, prefrontal cortex, and insula) (41). Higher levels of amyloid deposition in emotion-processing brain regions show a significant positive association with depressive symptom severity (42). Molecular mechanisms such as mitochondrial dysfunction (43), immune system (44), and HPA axis overactivation have been widely discussed as well (45). Notably, Gonzales et al. reported that individuals diagnosed with MCI who exhibited persistent subsyndromal depressive symptoms demonstrated significantly reduced level of CSF Aβ_1–42_, but their cognitive impairment was independent of Aβ and tau pathology (46). A separate large-scale cross-sectional investigation revealed that MCI patients exhibiting depressive symptoms were less likely to show amyloid pathology on testing, suggesting a lower burden of Aβ in this population (47), suggesting that other mechanisms such as neuroinflammation or functional network integration deficits may be more important. Functional imaging evidence indicates that depressive symptoms are closely associated with reduced global brain network integration efficiency and weakened connectivity in limbic systems (e.g., ACC-amygdala) (48). The shared neuropathological mechanisms described above provide a solid biological basis for the implementation of “integrated mental and cognitive health management” strategies at the public health level. Nevertheless, the underlying mechanisms are not fully understood, and conclusive evidence regarding the causality and precise biological pathways linking depressive symptoms to MCI is still lacking. Future studies need to integrate multimodal imaging and biomarkers for in-depth exploration.

The role of education as a protective cognitive resource has been further supported by numerous studies and may interact with multiple factors to influence the risk of MCI. Marselli et al. found that higher education was associated with greater cognitive reserve (CR), and that low CR was a significant risk factor for MCI (49). A systematic review by Corbo et al. indicated that high cognitive reserve (particularly accumulated through education and occupational activities) was associated with a reduced risk of MCI and could enhance cognitive performance in individuals with MCI (50). Yang et al. proposed the concept of “early-life cognitive reserve” and found that education was the most important factor explaining cognitive variation (contributing 43.9%), with a direct protective effect on cognitive function (51). These results suggest that education, by enhancing cognitive reserve, may serve as a moderating variable in the relationship linking depressive symptoms to MCI, improving neural compensation and cognitive flexibility, thereby delaying the clinical manifestation of cognitive decline. Furthermore, studies have indicated that cognitive reserve (including education, occupational complexity, and leisure activities) can mitigate the risk of MCI induced by (pre-)frailty, particularly among individuals with low cognitive reserve (52).

This study features several key methodological strengths. Firstly, it utilized a nationally representative, longitudinal cohort from CHARLS, offering a large sample size and extended follow-up period, which substantially enhanced the statistical power and generalizability of the results. Secondly, to mitigate potential reverse causality, we rigorously excluded individuals with pre-existing MCI at baseline. Furthermore, the analysis incorporated comprehensive adjustment for a wide range of potential confounding variables, thereby strengthening the validity of the observed associations. Finally, the robustness of the primary outcomes was confirmed through two complementary sensitivity analyses. Results from the stratified Cox model demonstrated that mild-to-moderate depressive symptoms remained significantly associated with an increased risk of MCI, although the risk estimate for the severe symptom group was attenuated and became non-significant, which may be attributable to reduced sample size in certain strata or differences in follow-up duration after stratification. Secondly, to account for competing risks (e.g., death), a multinomial logistic regression model was applied, yielding results highly consistent with the main analysis: the mild-to-moderate symptom group showed a significantly elevated risk of MCI, and severe symptoms also exhibited a marginally significant positive association. Although point estimates varied slightly across models, the overall trend remained consistent, further supporting the robustness of the finding that baseline depressive symptoms are tied to an increase in incident MCI risk, as well as the suggested dose-response relationship. However, several limitations should be noted. First, the CESD-10 scale, employed for evaluating depressive symptoms, does not capture variations across clinical subtypes of depression and does not establish a clinical diagnosis, serving solely as a symptomatological assessment. This limitation potentially obscures the impact of depressive heterogeneity on MCI risk. Second, the assessment was confined to depressive symptoms measured at a single timepoint and did not account for the potential influence of symptomatic fluctuations on cognitive trajectories. Third, our study period (2011-2020) overlapped with the COVID-19 pandemic. The pandemic, as a major external stressor, is known to have influenced mental health in the general population, including our study cohort. Although our primary analyses included the 2020 wave to maintain data completeness and statistical power, this may introduce bias if the pandemic differentially altered the reporting of depressive symptoms or its relationship with MCI incidence. We acknowledge this as a potential source of bias, as our design could not fully disentangle the specific effects of the pandemic from the long-term associations under investigation. Fourth, despite extensive adjustment for numerous covariates, the possibility of residual confounding remains, notably from unmeasured genetic variables like APOE ϵ4 status, which may introduce bias into the estimated relationship linking depressive symptoms to MCI. Finally, as the study population consisted of Chinese adults at midlife and beyond, the models and findings derived from this nationally representative Chinese cohort have not yet been validated in external populations, caution is warranted when generalizing the findings to other populations. Future studies should incorporate genetic data and multi-time point assessments of depressive symptoms to further elucidate causal pathways and moderating mechanisms between depressive symptoms and MCI.

## Conclusion

Using data from the CHARLS database, our analysis identifies baseline depressive symptoms as an independent risk factor for MCI development among Chinese adults aged 45 and above, even after adjusting for multiple potential confounders, with evidence of a monotonic dose-response relationship. Furthermore, we hypothesize that educational attainment may moderate this association, potentially through mechanisms such as enhancing cognitive reserve, health literacy, or access to resources. However, this observed relationship should be interpreted with caution, as it may be influenced by unmeasured confounding factors, such as childhood socioeconomic status or early-life cognitive ability. Future causal inference studies are needed to test this mechanism rigorously. These results highlight that screening for depressive symptoms should be incorporated into primary MCI prevention initiatives, particularly in populations with lower educational attainment. This provides important evidence for informing public health policies for MCI and dementia prevention worldwide. Furthermore, our findings underscore the need for future studies to not only screen for depressive symptoms but also to evaluate the effectiveness of interventions targeting these symptoms. Determining whether successful treatment of depression can translate into a reduced risk of subsequent MCI would be a critical next step, potentially revealing a modifiable pathway for primary prevention.

## Data Availability

Publicly available datasets were analyzed in this study. This data can be found here: https://charls.pku.edu.cn./.

## References

[1] Chen X Giles J Yao Y Yip W Meng Q Berkman L . The path to healthy ageing in China: a Peking University-Lancet Commission. Lancet. (2022) 400:1967–2006. doi: 10.1016/s0140-6736(22)01546-x, PMID: 36423650 PMC9801271

[2] Kim AB Arvanitakis Z . Insulin resistance, cognition, and Alzheimer disease. Obes (Silver Spring). (2023) 31:1486–98. doi: 10.1002/oby.23761, PMID: 37203336 PMC10421533

[3] Chen Y Bandosz P Stoye G Liu Y Wu Y Lobanov-Rostovsky S . Dementia incidence trend in England and Wales, 2002-19, and projection for dementia burden to 2040: analysis of data from the English Longitudinal Study of Ageing. Lancet Public Health. (2023) 8:e859–e67. doi: 10.1016/s2468-2667(23)00214-1, PMID: 37898518 PMC10958989

[4] Yang K Yang X Yin P Zhou M Tang Y . Temporal trend and attributable risk factors of Alzheimer’s disease and other dementias burden in China: Findings from the Global Burden of Disease Study 2021. Alzheimers Dement. (2024) 20:7871–84. doi: 10.1002/alz.14254, PMID: 39312279 PMC11567818

[5] Collaborators GDF . Estimation of the global prevalence of dementia in 2019 and forecasted prevalence in 2050: an analysis for the Global Burden of Disease Study 2019. Lancet Public Health. (2022) 7:e105–e25. doi: 10.1016/s2468-2667(21)00249-8, PMID: 34998485 PMC8810394

[6] Collaborators GNSD . Global, regional, and national burden of disorders affecting the nervous system, 1990-2021: a systematic analysis for the Global Burden of Disease Study 2021. Lancet Neurol. (2024) 23:344–81. doi: 10.1016/s1474-4422(24)00038-3, PMID: 38493795 PMC10949203

[7] Hendriks S Ranson JM Peetoom K Lourida I Tai XY de Vugt M . Risk factors for young-onset dementia in the UK biobank. JAMA Neurol. (2024) 81:134–42. doi: 10.1001/jamaneurol.2023.4929, PMID: 38147328 PMC10751655

[8] Jia L Quan M Fu Y Zhao T Li Y Wei C . Dementia in China: epidemiology, clinical management, and research advances. Lancet Neurol. (2020) 19:81–92. doi: 10.1016/s1474-4422(19)30290-x, PMID: 31494009

[9] Li X Feng X Sun X Hou N Han F Liu Y . Global, regional, and national burden of Alzheimer’s disease and other dementias, 1990-2019. Front Aging Neurosci. (2022) 14:937486. doi: 10.3389/fnagi.2022.937486, PMID: 36299608 PMC9588915

[10] Bai W Chen P Cai H Zhang Q Su Z Cheung T . Worldwide prevalence of mild cognitive impairment among community dwellers aged 50 years and older: a meta-analysis and systematic review of epidemiology studies. Age Ageing. (2022) 51:1-14. doi: 10.1093/ageing/afac173, PMID: 35977150

[11] Cong L Ren Y Wang Y Hou T Dong Y Han X . Mild cognitive impairment among rural-dwelling older adults in China: A community-based study. Alzheimers Dement. (2023) 19:56–66. doi: 10.1002/alz.12629, PMID: 35262288 PMC10078715

[12] Morris JC . Mild cognitive impairment and preclinical Alzheimer’s disease. Geriatrics. (2005) Suppl:9–14. 16025770

[13] Mitchell AJ Shiri-Feshki M . Rate of progression of mild cognitive impairment to dementia–meta-analysis of 41 robust inception cohort studies. Acta Psychiatr Scand. (2009) 119:252–65. doi: 10.1111/j.1600-0447.2008.01326.x, PMID: 19236314

[14] Jia L Du Y Chu L Zhang Z Li F Lyu D . Prevalence, risk factors, and management of dementia and mild cognitive impairment in adults aged 60 years or older in China: a cross-sectional study. Lancet Public Health. (2020) 5:e661–e71. doi: 10.1016/s2468-2667(20)30185-7, PMID: 33271079

[15] Leung DKY Chan WC Spector A Wong GHY . Prevalence of depression, anxiety, and apathy symptoms across dementia stages: A systematic review and meta-analysis. Int J Geriatr Psychiatry. (2021) 36:1330–44. doi: 10.1002/gps.5556, PMID: 33905138

[16] Elser H Horváth-Puhó E Gradus JL Smith ML Lash TL Glymour MM . Association of early-, middle-, and late-life depression with incident dementia in a Danish cohort. JAMA Neurol. (2023) 80:949–58. doi: 10.1001/jamaneurol.2023.2309, PMID: 37486689 PMC10366950

[17] Huang YY Gan YH Yang L Cheng W Yu JT . Depression in alzheimer’s disease: epidemiology, mechanisms, and treatment. Biol Psychiatry. (2024) 95:992–1005. doi: 10.1016/j.biopsych.2023.10.008, PMID: 37866486

[18] Pink A Krell-Roesch J Syrjanen JA Vassilaki M Lowe VJ Vemuri P . A longitudinal investigation of Aβ, anxiety, depression, and mild cognitive impairment. Alzheimers Dement. (2022) 18:1824–31. doi: 10.1002/alz.12504, PMID: 34877794 PMC9174347

[19] Wei C Zhao J Hu R Wei X . Association between depressive status and mild cognitive impairment in middle-aged and elderly Chinese adults from CHARLS study. Front Psychiatry. (2025) 16:1516341. doi: 10.3389/fpsyt.2025.1516341, PMID: 40018684 PMC11865024

[20] Han FF Wang HX Wu JJ Yao W Hao CF Pei JJ . Depressive symptoms and cognitive impairment: A 10-year follow-up study from the Survey of Health, Ageing and Retirement in Europe. Eur Psychiatry. (2021) 64:e55. doi: 10.1192/j.eurpsy.2021.2230, PMID: 34446123 PMC8446071

[21] Zhao Y Hu Y Smith JP Strauss J Yang G . Cohort profile: the China health and retirement longitudinal study (CHARLS). Int J Epidemiol. (2014) 43:61–8. doi: 10.1093/ije/dys203, PMID: 23243115 PMC3937970

[22] von Elm E Altman DG Egger M Pocock SJ Gøtzsche PC Vandenbroucke JP . The Strengthening the Reporting of Observational Studies in Epidemiology (STROBE) statement: guidelines for reporting observational studies. Lancet. (2007) 370:1453–7. doi: 10.1016/s0140-6736(07)61602-x, PMID: 18064739

[23] Chen H Mui AC . Factorial validity of the Center for Epidemiologic Studies Depression Scale short form in older population in China. Int Psychogeriatr. (2014) 26:49–57. doi: 10.1017/s1041610213001701, PMID: 24125553

[24] Sang N Liu RC Zhang MH Lu ZX Wu ZG Zhang MY . Changes in frailty and depressive symptoms among middle-aged and older Chinese people: a nationwide cohort study. BMC Public Health. (2024) 24:301. doi: 10.1186/s12889-024-17824-3, PMID: 38273230 PMC10811919

[25] Chen R Chen Q Lu G Zhang M Zhang M Yang H . Sleep duration and depressive symptoms in Chinese middle-aged and older adults: The moderating effects of grip strength. J Affect Disord. (2023) 339:348–54. doi: 10.1016/j.jad.2023.07.059, PMID: 37451435

[26] Yang X Pan A Gong J Wen Y Ye Y Wu JH . Prospective associations between depressive symptoms and cognitive functions in middle-aged and elderly Chinese adults. J Affect Disord. (2020) 263:692–7. doi: 10.1016/j.jad.2019.11.048, PMID: 31744738

[27] Zhang Z He P Liu M Zhou C Liu C Li H . Association of depressive symptoms with rapid kidney function decline in adults with normal kidney function. Clin J Am Soc Nephrol. (2021) 16:889–97. doi: 10.2215/cjn.18441120, PMID: 34052796 PMC8216614

[28] Cui Y Xu Z Cui Z Guo Y Wu P Zhou X . Comparative study of insulin resistance surrogate indices to predict mild cognitive impairment among Chinese non-diabetic adults. Lipids Health Dis. (2024) 23:357. doi: 10.1186/s12944-024-02353-0, PMID: 39487494 PMC11529243

[29] Zhang L Chen W Miao H Zou T Xiang X Wu R . Association between physical activity levels and mild cognitive impairment in Chinese older adults: a cross-sectional study from the China health and retirement longitudinal study. Front Public Health. (2025) 13:1564544. doi: 10.3389/fpubh.2025.1564544, PMID: 40255385 PMC12006155

[30] Hu Y Peng W Ren R Wang Y Wang G . Sarcopenia and mild cognitive impairment among elderly adults: The first longitudinal evidence from CHARLS. J Cachexia Sarcopenia Muscle. (2022) 13:2944–52. doi: 10.1002/jcsm.13081, PMID: 36058563 PMC9745544

[31] Richards M Touchon J Ledesert B Richie K . Cognitive decline in ageing: are AAMI and AACD distinct entities? Int J Geriatr Psychiatry. (1999) 14:534–40. doi: 10.1002/(sici)1099-1166(199907)14:7<534::aid-gps963>3.0.co;2-b, PMID: 10440973

[32] Zhang D Zhou Y Liu Y Wu S . Association between residential environment quality with mild cognitive impairment among middle and elderly adults in China. J Neurol Sci. (2024) 467:123318. doi: 10.1016/j.jns.2024.123318, PMID: 39608295

[33] Li F Wang Y Shi B Sun S Wang S Pang S . Association between the cumulative average triglyceride glucose-body mass index and cardiovascular disease incidence among the middle-aged and older population: a prospective nationwide cohort study in China. Cardiovasc Diabetol. (2024) 23:16. doi: 10.1186/s12933-023-02114-w, PMID: 38184577 PMC10771655

[34] Chen L Li D Tang K Li Z Xiaoyun H . Sleep duration and leisure activities are involved in regulating the association of depressive symptoms, muscle strength, physical function and mild cognitive impairment. Heliyon. (2024) 10:e33832. doi: 10.1016/j.heliyon.2024.e33832, PMID: 39027538 PMC11255586

[35] Zhou S Wang Q Zhang J Wang Q Hou F Han X . Depressive symptoms and cognitive decline among Chinese rural elderly individuals: A longitudinal study with 2-year follow-up. Front Public Health. (2022) 10:939150. doi: 10.3389/fpubh.2022.939150, PMID: 35910927 PMC9326072

[36] Du Y Hu N Yu Z Liu X Ma Y Li J . Characteristics of the cognitive function transition and influencing factors among Chinese older people: An 8-year longitudinal study. J Affect Disord. (2023) 324:433–9. doi: 10.1016/j.jad.2022.12.116, PMID: 36586609

[37] Lee CH Kim DH Moon YS . Differential associations between depression and cognitive function in MCI and AD: a cross-sectional study. Int Psychogeriatr. (2019) 31:1151–8. doi: 10.1017/s1041610218001527, PMID: 30696505

[38] Manly JJ Jones RN Langa KM Ryan LH Levine DA McCammon R . Estimating the prevalence of dementia and mild cognitive impairment in the US: the 2016 health and retirement study harmonized cognitive assessment protocol project. JAMA Neurol. (2022) 79:1242–9. doi: 10.1001/jamaneurol.2022.3543, PMID: 36279130 PMC9593315

[39] Kulshreshtha A Parker ES Fowler NR Summanwar D Ben Miled Z Owora AH . Prevalence of unrecognized cognitive impairment in federally qualified health centers. JAMA Netw Open. (2024) 7:e2440411. doi: 10.1001/jamanetworkopen.2024.40411, PMID: 39436648 PMC11581540

[40] Jannati A Toro-Serey C Gomes-Osman J Banks R Ciesla M Showalter J . Digital Clock and Recall is superior to the Mini-Mental State Examination for the detection of mild cognitive impairment and mild dementia. Alzheimers Res Ther. (2024) 16:2. doi: 10.1186/s13195-023-01367-7, PMID: 38167251 PMC10759368

[41] Zacková L Jáni M Brázdil M Nikolova YS Marečková K . Cognitive impairment and depression: Meta-analysis of structural magnetic resonance imaging studies. NeuroImage Clin. (2021) 32:102830. doi: 10.1016/j.nicl.2021.102830, PMID: 34560530 PMC8473769

[42] Brendel M Pogarell O Xiong G Delker A Bartenstein P Rominger A . Depressive symptoms accelerate cognitive decline in amyloid-positive MCI patients. Eur J Nucl Med Mol Imaging. (2015) 42:716–24. doi: 10.1007/s00259-014-2975-4, PMID: 25631614 PMC5849231

[43] Choi J Beroncal EL Chernega T Brooks HJ Kennedy JL Fisher CE . Exploring mitochondrial blood-based and genetic markers in older adults with mild cognitive impairment and remitted major depressive disorder. Transl Psychiatry. (2024) 14:457. doi: 10.1038/s41398-024-03155-9, PMID: 39468012 PMC11519657

[44] Hayley S Hakim AM Albert PR . Depression, dementia and immune dysregulation. Brain. (2021) 144:746–60. doi: 10.1093/brain/awaa405, PMID: 33279966 PMC8041341

[45] Galts CPC Bettio LEB Jewett DC Yang CC Brocardo PS Rodrigues ALS . Depression in neurodegenerative diseases: Common mechanisms and current treatment options. Neurosci Biobehav Rev. (2019) 102:56–84. doi: 10.1016/j.neubiorev.2019.04.002, PMID: 30995512

[46] Gonzales MM Insel PS Nelson C Tosun D Schöll M Mattsson N . Chronic depressive symptomatology and CSF amyloid beta and tau levels in mild cognitive impairment. Int J Geriatr Psychiatry. (2018) 33:1305–11. doi: 10.1002/gps.4926, PMID: 29953668

[47] Wiels WA Oomens JE Engelborghs S Baeken C von Arnim CAF Boada M . Depressive symptoms and amyloid pathology. JAMA Psychiatry. (2025) 82:296–310. doi: 10.1001/jamapsychiatry.2024.4305, PMID: 39841452 PMC11883504

[48] Csukly G Tombor L Hidasi Z Csibri E Fullajtár M Huszár Z . Low Functional network integrity in cognitively unimpaired and MCI subjects with depressive symptoms: results from a multi-center fMRI study. Transl Psychiatry. (2024) 14:179. doi: 10.1038/s41398-024-02891-2, PMID: 38580625 PMC10997664

[49] Marselli G Favieri F Forte G Corbo I Agostini F Guarino A . The protective role of cognitive reserve: an empirical study in mild cognitive impairment. BMC Psychol. (2024) 12:334. doi: 10.1186/s40359-024-01831-5, PMID: 38849930 PMC11157959

[50] Corbo I Marselli G Di Ciero V Casagrande M . The protective role of cognitive reserve in mild cognitive impairment: A systematic review. J Clin Med. (2023) 12:1-16. doi: 10.3390/jcm12051759, PMID: 36902545 PMC10002518

[51] Yang Y Chen Y Yang C Chen K Li X Zhang Z . Contributions of early-life cognitive reserve and late-life leisure activity to successful and pathological cognitive aging. BMC Geriatr. (2022) 22:831. doi: 10.1186/s12877-022-03530-5, PMID: 36319960 PMC9628084

[52] Li Y Liu Q Si H Zhou W Yu J Bian Y . Effects of (pre)frailty and cognitive reserve on mild cognitive impairment among community-dwelling older adults. Arch Gerontol Geriatr. (2024) 126:105533. doi: 10.1016/j.archger.2024.105533, PMID: 38878599

