# Supplementary material for: The role of elevated depressive symptoms in the incident mild cognitive impairment in China: a 9-year prospective cohort study of middle-aged and older Chinese adults (2011–2020)
Source: Front Psychiatry. 2025 Dec 9;16:1726680. doi: 10.3389/fpsyt.2025.1726680 (PMC12723517; doi:10.3389/fpsyt.2025.1726680)
Supplement: Supplementary file 1 [file Supplementaryfile1.docx]

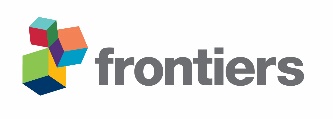
 Supplementary Material

Supplementary TABLE S1**.** Baseline characteristics of participants overall and by binary depressive symptom classification

| **Characteristics** |  | **Overall** | **Clinically Relevant Dichotomization** | | ***p*-value** |
| --- | --- | --- | --- | --- | --- |
|  |  |  | **No Elevated Symptoms** | **Elevated Symptoms** |  |
| **N** |  | 9461 | 6636 | 2825 |  |
| **Age, years** |  | 58.16 (9.02) | 57.80 (9.00) | 59.00 (9.00) | <0.001 |
| **Gender** | Male | 5203 (55.0) | 3878 (58.4) | 1325 (46.9) | <0.001 |
|  | Female | 4258 (45.0) | 2758 (41.6) | 1500 (53.1) |  |
| **Marital status** | Unmarried | 1354 (14.3) | 795 (12.0) | 559 (19.8) | <0.001 |
|  | Married | 8107 (85.7) | 5841 (88.0) | 2266 (80.2) |  |
| **Education level** | Elementary school or below | 5211 (55.1) | 3363 (50.7) | 1848 (65.4) | <0.001 |
|  | Middle school | 3626 (38.3) | 2732 (41.2) | 894 (31.6) |  |
|  | College or above | 624 (6.6) | 541 ( 8.2) | 83 (2.9) |  |
| **Residence** | Urban | 2763 (29.2) | 2172 ( 32.7) | 591 (20.9) | <0.001 |
|  | Rural | 6698 (70.8) | 4464 (67.3) | 2234 (79.1) |  |
| **BMI, kg/m2** |  | 24.20 (26.62) | 24.57 (31.68) | 23.33 (3.78) | 0.037 |
| **Waist** |  | 85.15 (12.55) | 85.58 (12.61) | 84.15 (12.34) | <0.001 |
| **Weight** |  | 61.09 (11.88) | 62.22 (11.92) | 58.41 (11.35) | <0.001 |
| **Height** |  | 1.60 (0.09) | 1.61 (0.09) | 1.58 (0.08) | <0.001 |
| **SBP, mmHg** |  | 129.26 (20.80) | 129.50 (20.58) | 128.71 (21.31) | 0.092 |
| **DBP, mmHg** |  | 75.92 (12.18) | 76.26 (12.15) | 75.12 (12.21) | <0.001 |
| **Smoking status** | Current | 3194 (33.8) | 2282 (34.4) | 912 (32.3) | 0.002 |
|  | Former | 977 (10.3) | 717 (10.8) | 260 (9.2) |  |
|  | Never | 5290 (55.9) | 3637 (54.8) | 1653 (58.5) |  |
| **Drinking status** | Current | 3233 (34.2) | 2431 (36.6) | 802 (28.4) | <0.001 |
|  | Former | 806 (8.5) | 508 (7.7) | 298 (10.5) |  |
|  | Never | 5422 (57.3) | 3697 (55.7) | 1725 (61.1) |  |
| **Nighttime sleep duration** |  | 33.25 (42.21) | 34.55 (42.75) | 30.21 (40.78) | <0.001 |
| **Dyslipidemia** | No | 8374 (88.5) | 5900 (88.9) | 2474 (87.6) | 0.068 |
|  | Yes | 1087 (11.5) | 736 (11.1) | 351 (12.4) |  |
| **Hypertension** | No | 6961 (73.6) | 4965 (74.8) | 1996 (70.7) | <0.001 |
|  | Yes | 2500 (26.4) | 1671 (25.2) | 829 (29.3) |  |
| **Diabetes** | No | 8839 (93.4) | 6228 (93.9) | 2611 (92.4) | 0.012 |
|  | Yes | 622 (6.6) | 408 (6.1) | 214 (7.6) |  |
| **Kidney disease** | No | 8920 (94.3) | 6338 (95.5) | 2582 (91.4) | <0.001 |
|  | Yes | 541 (5.7) | 298 (4.5) | 243 (8.6) |  |
| **Liver disease** | No | 9110 (96.3) | 6419 (96.7) | 2691 (95.3) | 0.001 |
|  | Yes | 351 (3.7) | 217 (3.3) | 134 (4.7) |  |
| **Cardiovascular disease** | No | 8242 (87.1) | 5902 (88.9) | 2340 (82.8) | <0.001 |
|  | Yes | 1219 (12.9) | 734 ( 11.1) | 485 (17.2) |  |
| **Stroke** | No | 9252 (97.8) | 6530 (98.4) | 2722 (96.4) | <0.001 |
|  | Yes | 209 (2.2) | 106 (1.6) | 103 (3.6) |  |
| **Lipid-lowering treatment** | No | 8941 (94.5) | 6300 (94.9) | 2641 (93.5) | 0.005 |
|  | Yes | 520 (5.5) | 336 (5.1) | 184 (6.5) |  |
| **Antihypertensive treatment** | No | 7582 (80.1) | 5389 (81.2) | 2193 (77.6) | <0.001 |
|  | Yes | 1879 (19.9) | 1247 (18.8) | 632 (22.4) |  |
| **Hypoglycemic treatment** | No | 9036 (95.5) | 6363 (95.9) | 2673 (94.6) | 0.008 |
|  | Yes | 425 (4.5) | 273 (4.1) | 152 (5.4) |  |
| **Cardiovascular therapy** | No | 8704 (92.0) | 6215 (93.7) | 2489 (88.1) | <0.001 |
|  | Yes | 757 (8.0) | 421 (6.3) | 336 (11.9) |  |
| **Stroke therapy** | No | 9349 (98.8) | 6580 (99.2) | 2769 (98.0) | <0.001 |
|  | Yes | 112 (1.2) | 56 (0.8) | 56 (2.0) |  |
| **HbA1c, %** |  | 5.25 (0.82) | 5.24 (0.79) | 5.30 (0.89) | 0.001 |
| **FBG, mg/dL** |  | 110.40 (37.93) | 110.02 (36.07) | 111.30 (41.97) | 0.135 |
| **TG, mg/dL** |  | 136.86 (101.76) | 138.56 (105.00) | 132.88 (93.59) | 0.013 |
| **TC, mg/dL** |  | 192.69 (38.60) | 192.25 (38.53) | 193.73 (38.75) | 0.086 |
| **HDL-c, mg/dL** |  | 49.85 (15.13) | 49.43 (15.08) | 50.82 (15.21) | <0.001 |
| **LDL-c, mg/dL** |  | 116.24 (35.68) | 115.76 (35.80) | 117.37 (35.40) | 0.045 |
| **BUN, mg/dL** |  | 15.62 (4.39) | 15.68 (4.40) | 15.47 (4.38) | 0.030 |
| **UA, mg/dL** |  | 4.59 (1.28) | 4.66 (1.29) | 4.42 (1.24) | <0.001 |
| **CRP, mg/dL** |  | 2.80 (7.77) | 2.71 (7.60) | 3.02 (8.16) | 0.076 |
| **CESD score** |  | 7.33 (5.83) | 4.19 (2.77) | 14.72 (4.26) | <0.001 |
| **Cognition score** |  | 17.52 (3.67) | 18.00 (3.68) | 16.39 (3.41) | <0.001 |

Note:Data are presented as mean (standard deviation) for continuous variables and n (%) for categorical variables. P-values were derived from T-test for continuous variables and the chi-square test for categorical variables across the three depressive symptom groups.

Abbreviations: BMI, body mass index; SBP, systolic blood pressure; DBP, diastolic blood pressure; HbA1c, glycosylated hemoglobin; FBG, fasting blood glucose; TG, triglycerides; TC, total cholesterol; HDL-c, high-density lipoprotein cholesterol; LDL-c, low-density lipoprotein cholesterol; BUN, blood urea nitrogen; UA, uric acid; CRP, C-reactive protein

Supplementary TABLE S2**.** Baseline characteristics of participants overall and by incident MCI status

| **Characteristics** |  | **Overall** | **Outcome** | | ***p*-value** |
| --- | --- | --- | --- | --- | --- |
|  |  |  | **No MCI** | **MCI** |  |
| **N（%）** |  | 9461 | 8185 | 1276 |  |
| **Age, years** |  | 58.16 (9.02) | 58.25 (9.11) | 57.59 (8.37) | 0.016 |
| **Gender** | Male | 5203 (55.0) | 4575 (55.9) | 628 (49.2) | <0.001 |
|  | Female | 4258 (45.0) | 3610 (44.1) | 648 (50.8) |  |
| **Marital status** | Unmarried | 1354 (14.3) | 1151 (14.1) | 203 (15.9) | 0.087 |
|  | Married | 8107 (85.7) | 7034 (85.9) | 1073 (84.1) |  |
| **Education level** | Elementary school or below | 5211 (55.1) | 608 (7.4) | 16 (1.3) | <0.001 |
|  | Middle school | 3626 (38.3) | 4257 (52.0) | 954 (74.8) |  |
|  | College or above | 624 ( 6.6) | 3320 (40.6) | 306 (24.0) |  |
| **Residence** | Urban | 2763 (29.2) | 2597 (31.7) | 166 (13.0) | <0.001 |
|  | Rural | 6698 (70.8) | 5588 (68.3) | 1110 (87.0) |  |
| **BMI, kg/m2** |  | 24.20 (26.62) | 24.29 (28.58) | 23.60 (3.81) | 0.388 |
| **Waist** |  | 85.15 (12.55) | 85.23 (12.64) | 84.64 (11.91) | 0.115 |
| **Weight** |  | 61.09 (11.88) | 61.39 (11.92) | 59.12 (11.43) | <0.001 |
| **Height** |  | 1.60 (0.09) | 1.60 (0.09) | 1.58 (0.08) | <0.001 |
| **SBP, mmHg** |  | 129.26 (20.80) | 129.39 (20.66) | 128.45 (21.67) | 0.130 |
| **DBP, mmHg** |  | 75.92 (12.18) | 76.02 (12.12) | 75.26 (12.55) | 0.037 |
| **Smoking status** | Current | 3194 (33.8) | 2765 (33.8) | 429 (33.6) | 0.022 |
|  | Former | 977 (10.3) | 872 (10.7) | 105 (8.2) |  |
|  | Never | 5290 (55.9) | 4548 (55.6) | 742 (58.2) |  |
| **Drinking status** | Current | 3233 (34.2) | 2831 (34.6) | 402 (31.5) | 0.096 |
|  | Former | 806 ( 8.5) | 694 (8.5) | 112 (8.8) |  |
|  | Never | 5422 (57.3) | 4660 (56.9) | 762 (59.7) |  |
| **Nighttime sleep duration** |  | 33.25 (42.21) | 6.47 (1.70) | 6.41 (1.87) | 0.287 |
| **Dyslipidemia** | No | 8374 (88.5) | 7213 (88.1) | 1161 (91.0) | 0.003 |
|  | Yes | 1087 (11.5) | 972 (11.9) | 115 (9.0) |  |
| **Hypertension** | No | 6961 (73.6) | 5997 (73.3) | 964 (75.5) | 0.092 |
|  | Yes | 2500 (26.4) | 2188 (26.7) | 312 (24.5) |  |
| **Diabetes** | No | 8839 (93.4) | 7645 (93.4) | 1194 (93.6) | 0.866 |
|  | Yes | 622 ( 6.6) | 540 (6.6) | 82 (6.4) |  |
| **Kidney disease** | No | 8920 (94.3) | 7729 (94.4) | 1191 (93.3) | 0.135 |
|  | Yes | 541 (5.7) | 456 (5.6) | 85 (6.7) |  |
| **Liver disease** | No | 9110 (96.3) | 7882 (96.3) | 1228 (96.2) | 0.980 |
|  | Yes | 351 (3.7) | 303 (3.7) | 48 (3.8) |  |
| **Cardiovascular disease** | No | 8242 (87.1) | 7104 (86.8) | 1138 (89.2) | 0.020 |
|  | Yes | 1219 (12.9) | 1081 (13.2) | 138 (10.8) |  |
| **Stroke** | No | 9252 (97.8) | 8002 (97.8) | 1250 (98.0) | 0.730 |
|  | Yes | 209 (2.2) | 183 (2.2) | 26 (2.0) |  |
| **Lipid-lowering treatment** | No | 8941 (94.5) | 7726 (94.4) | 1215 (95.2) | 0.254 |
|  | Yes | 520 (5.5) | 459 (5.6) | 61 (4.8) |  |
| **Antihypertensive treatment** | No | 7582 (80.1) | 6525 (79.7) | 1057 (82.8) | 0.010 |
|  | Yes | 1879 (19.9) | 1660 (20.3) | 219 (17.2) |  |
| **Hypoglycemic treatment** | No | 9036 (95.5) | 7811 (95.4) | 1225 (96.0) | 0.398 |
|  | Yes | 425 (4.5) | 374 (4.6) | 51 (4.0) |  |
| **Cardiovascular therapy** | No | 8704 (92.0) | 7517 (91.8) | 1187 (93.0) | 0.162 |
|  | Yes | 757 ( 8.0) | 668 (8.2) | 89 (7.0) |  |
| **Stroke therapy** | No | 9349 (98.8) | 8085 (98.8) | 1264 (99.1) | 0.468 |
|  | Yes | 112 (1.2) | 100 (1.2) | 12 (0.9) |  |
| **HbA1c, %** |  | 5.25 (0.82) | 5.25 (0.81) | 5.28 (0.85) | 0.182 |
| **FBG, mg/dL** |  | 110.40 (37.93) | 110.41 (37.60) | 110.35 (40.00) | 0.957 |
| **TG, mg/dL** |  | 136.86 (101.76) | 136.97 (100.51) | 136.19 (109.47) | 0.798 |
| **TC, mg/dL** |  | 192.69 (38.60) | 192.51 (38.78) | 193.87 (37.38) | 0.242 |
| **HDL-c, mg/dL** |  | 49.85 (15.13) | 49.64 (15.12) | 51.18 (15.16) | 0.001 |
| **LDL-c, mg/dL** |  | 116.24 (35.68) | 116.30 (35.71) | 115.83 (35.52) | 0.659 |
| **BUN, mg/dL** |  | 15.62 (4.39) | 15.59 (4.38) | 15.77 (4.46) | 0.191 |
| **UA, mg/dL** |  | 4.59 (1.28) | 4.61 (1.28) | 4.46 (1.27) | <0.001 |
| **CRP, mg/dL** |  | 2.80 (7.77) | 2.75 (7.64) | 3.11 (8.55) | 0.128 |
| **CESD score** |  | 7.33 (5.83) | 7.14 (5.75) | 8.58 (6.17) | <0.001 |
| **Cognition score** |  | 17.52 (3.67) | 17.76 (3.69) | 15.97 (3.18) | <0.001 |

Note:Data are presented as mean (standard deviation) for continuous variables and n (%) for categorical variables. P-values were derived from T-test for continuous variables and the chi-square test for categorical variables across the three depressive symptom groups.

Abbreviations: BMI, body mass index; SBP, systolic blood pressure; DBP, diastolic blood pressure; HbA1c, glycosylated hemoglobin; FBG, fasting blood glucose; TG, triglycerides; TC, total cholesterol; HDL-c, high-density lipoprotein cholesterol; LDL-c, low-density lipoprotein cholesterol; BUN, blood urea nitrogen; UA, uric acid; CRP, C-reactive protein

Supplementary TABLE S3. **Missing data patterns of baseline variables**

| **Variable** | **Count** |
| --- | --- |
| Gender | 0 |
| Marital status | 0 |
| Education level | 0 |
| Residence | 0 |
| Smoking status | 0 |
| Drinking status | 0.000422788 |
| Dyslipidemia | 0.013529225 |
| Lipid-lowering treatment | 0.014057711 |
| Hypertension | 0.002748124 |
| Antihypertensive treatment | 0.002959518 |
| Diabetes | 0.006553219 |
| Hypoglycemic treatment | 0.006658916 |
| Cardiovascular disease | 0.003910792 |
| Cardioprotective therapy | 0.004439277 |
| Stroke | 0.001374062 |
| Cerebroprotective therapy | 0.001374062 |
| Kidney disease | 0.004016489 |
| Liver disease | 0.005601945 |
| Age | 0 |
| BMI | 0.161188035 |
| Waist | 0.155374696 |
| SBP | 0.158334214 |
| DBP | 0.158334214 |
| Nighttime sleep duration | 0.002853821 |
| HbA1c | 0.302610718 |
| FBG | 0.306838601 |
| TG | 0.305675933 |
| TC | 0.30578163 |
| HDL-c | 0.305358842 |
| LDL-c | 0.306204418 |
| BUN | 0.305464539 |
| UA | 0.305147447 |
| CRP | 0.305147447 |

Abbreviations: BMI, body mass index; SBP, systolic blood pressure; DBP, diastolic blood pressure; HbA1c, glycosylated hemoglobin; FBG, fasting blood glucose; TG, triglycerides; TC, total cholesterol; HDL-c, high-density lipoprotein cholesterol; LDL-c, low-density lipoprotein cholesterol; BUN, blood urea nitrogen; UA, uric acid; CRP, C-reactive protein

Supplementary TABLE S4. Baseline characteristics of participants overall and by severity of depressive symptoms: before multiple imputation

| **Characteristics** | **Overall** | **Symptom Severity Stratification** | | | ***p*-value** |
| --- | --- | --- | --- | --- | --- |
|  |  | **No Elevated Symptoms** | **Mild-Moderate Symptoms** | **Severe Symptoms** |  |
| **N** | 9461 | 6636 | 2494 | 331 |  |
| **Age, years** | 58.16 (9.02) | 57.80(9.00) | 58.89(9.04) | 59.80(8.68) | <0.001 |
| **Gender** |  |  |  |  |  |
| Male | 5203 (55.0) | 3878(58.4) | 1175(47.1) | 150(45.3) | <0.001 |
| Female | 4258 (45.0) | 2758(41.6) | 1319(52.9) | 181(54.7) |  |
| **Marital status** |  |  |  |  |  |
| Unmarried | 1354 (14.3) | 795(12.0) | 484(19.4) | 75(22.7) | <0.001 |
| Married | 8107 (85.7) | 5841(88.0) | 2010(80.6) | 256(77.3) |  |
| **Education level** |  |  |  |  |  |
| Elementary school or below | 5211 (55.1) | 3363(50.7) | 1609(64.5) | 239(72.2) | <0.001 |
| Middle school | 3626 (38.3) | 2732(41.2) | 806(32.3) | 88(26.6) |  |
| College or above | 624 ( 6.6) | 541(8.2) | 79(3.2) | 4(1.2) |  |
| **Residence** |  |  |  |  |  |
| Urban | 2763 (29.2) | 2172(32.7) | 549(22.0) | 42(12.7) | <0.001 |
| Rural | 6698 (70.8) | 4464(67.3) | 1945(78.0) | 289(87.3) |  |
| **BMI, kg/m2** | 24.24 (29.01) | 24.67(34.69) | 23.26(3.78) | 23.22(3.76) | 0.136 |
| **Waist** | 84.98 (12.49) | 85.42(12.55) | 84.05(12.06) | 83.38(14.03) | <0.001 |
| **Weight** | 60.76 (11.62) | 61.88(11.67) | 58.20(11.08) | 58.13(11.22) | <0.001 |
| **Height** | 1.60 (0.09) | 1.60(0.09) | 1.58(0.08) | 1.58(0.08) | <0.001 |
| **SBP, mmHg** | 129.15 (20.78) | 129.42(20.46) | 128.40(21.25) | 129.63(23.21) | 0.147 |
| **DBP, mmHg** | 75.79 (12.16) | 76.14(12.08) | 74.94(12.18) | 75.48(13.07) | 0.001 |
| **Smoking status** |  |  |  |  |  |
| Current | 3194 (33.8) | 2282(34.4) | 798(32.0) | 114(34.4) | 0.010 |
| Former | 977 (10.3) | 717(10.8) | 231(9.3) | 29(8.8) |  |
| Never | 5290 (55.9) | 3637(54.8) | 1465(58.7) | 188(56.8) |  |
| **Drinking status** |  |  |  |  |  |
| Current | 3232 (34.2) | 2431(36.6) | 719(28.9) | 82(24.8) | <0.001 |
| Former | 806 ( 8.5) | 508(7.7) | 251(10.1) | 47(14.2) |  |
| Never | 5419 (57.3) | 3696(55.7) | 1522(61.1) | 201(60.9) |  |
| **Nighttime sleep duration** | 6.46 (1.73) | 6.72(1.56) | 5.93(1.91) | 5.24(1.96) | <0.001 |
| **Dyslipidemia** |  |  |  |  |  |
| No | 8266 (88.6) | 5828(89.0) | 2155(87.6) | 283(87.3) | 0.126 |
| Yes | 1067 (11.4) | 720(11.0) | 306(12.4) | 41(12.7) |  |
| **Hypertension** |  |  |  |  |  |
| No | 6943 (73.6) | 4954(74.8) | 1771(71.3) | 218(66.3) | <0.001 |
| Yes | 2492 (26.4) | 1667(25.2) | 714(28.7) | 111(33.7) |  |
| **Diabetes** |  |  |  |  |  |
| No | 8780 (93.4) | 6187(93.9) | 2291(92.5) | 302(91.5) | 0.024 |
| Yes | 619 ( 6.6) | 405(6.1) | 186(7.5) | 28(8.5) |  |
| **Kidney disease** |  |  |  |  |  |
| No | 8884 (94.3) | 6314(95.5) | 2292(92.2) | 278(85.0) | <0.001 |
| Yes | 539 ( 5.7) | 297(4.5) | 193(7.8) | 49(15.0) |  |
| **Liver disease** |  |  |  |  |  |
| No | 9059 (96.3) | 6389(96.7) | 2367(95.6) | 303(92.4) | <0.001 |
| Yes | 349 ( 3.7) | 215(3.3) | 109(4.4) | 25(7.6) |  |
| **Heart disease** |  |  |  |  |  |
| No | 8210 (87.1) | 5883(88.9) | 2069(83.5) | 258(78.2) | <0.001 |
| Yes | 1214 (12.9) | 733(11.1) | 409(16.5) | 72(21.8) |  |
| **Stroke** |  |  |  |  |  |
| No | 9239 (97.8) | 6521(98.4) | 2406(96.6) | 312(94.3) | <0.001 |
| Yes | 209 ( 2.2) | 106(1.6) | 84(3.4) | 19(5.7) |  |
| **Lipid-lowering treatment** |  |  |  |  |  |
| No | 8817 (94.5) | 6214(95.0) | 2302(93.6) | 301(92.9) | 0.016 |
| Yes | 511 ( 5.5) | 330(5.0) | 158(6.4) | 23(7.1) |  |
| **Antihypertensive treatment** |  |  |  |  |  |
| No | 7557 (80.1) | 5374(81.2) | 1931(77.7) | 252(76.6) | <0.001 |
| Yes | 1876 (19.9) | 1245(18.8) | 554(22.3) | 77(23.4) |  |
| **Hypoglycemic treatment** |  |  |  |  |  |
| No | 8976 (95.5) | 6321(95.9) | 2343(94.6) | 312(94.5) | 0.018 |
| Yes | 422 ( 4.5) | 270(4.1) | 134(5.4) | 18(5.5) |  |
| **Cardioprotective therapy** |  |  |  |  |  |
| No | 8666 (92.0) | 6193(93.7) | 2199(88.7) | 274(83.3) | <0.001 |
| Yes | 753 ( 8.0) | 419(6.3) | 279(11.3) | 55(16.7) |  |
| **Cerebroprotective therapy** |  |  |  |  |  |
| No | 9336 (98.8) | 6571(99.2) | 2445(98.2) | 320(96.7) | <0.001 |
| Yes | 112 ( 1.2) | 56(0.8) | 45(1.8) | 11(3.3) |  |
| **HbA1c, %** | 5.26 (0.81) | 5.25(0.78) | 5.28(0.85) | 5.27(0.97) | 0.232 |
| **FBG, mg/dL** | 110.44 (37.71) | 110.24(36.50) | 111.13(40.59) | 108.98(37.88) | 0.575 |
| **TG, mg/dL** | 136.06 (107.91) | 138.16(112.20) | 131.18(94.53) | 133.52(118.10) | 0.063 |
| **TC, mg/dL** | 193.33 (38.17) | 193.07(38.30) | 194.24(37.98) | 191.40(37.13) | 0.391 |
| **HDL-c, mg/dL** | 50.18 (15.08) | 49.72(15.12) | 51.36(15.03) | 50.03(14.51) | <0.001 |
| **LDL-c, mg/dL** | 116.83 (34.88) | 116.59(35.13) | 117.66(34.64) | 115.38(32.18) | 0.434 |
| **BUN** | 15.63 (4.39) | 15.69(4.42) | 15.53(4.29) | 15.30(4.45) | 0.196 |
| **UA** | 4.57 (1.28) | 4.63(1.28) | 4.43(1.27) | 4.28(1.11) | <0.001 |
| **CRP** | 2.72 (7.32) | 2.61(6.75) | 3.03(8.81) | 2.55(5.12) | 0.105 |
| **CESD score** | 7.33 (5.83) | 4.19(2.77) | 13.58(2.96) | 23.31(2.21) | <0.001 |
| **Cognition score** | 17.52 (3.67) | 18.00 (3.68) | 16.49 (3.38) | 15.64 (3.52) | <0.001 |

**Note: Data are presented as mean (standard deviation) for continuous variables and n (%) for categorical variables. P-values were derived from one-way ANOVA for continuous variables and the chi-square test for categorical variables across the three depressive symptom groups.**

**Abbreviations: BMI, body mass index; SBP, systolic blood pressure; DBP, diastolic blood pressure; HbA1c, glycosylated hemoglobin; FBG, fasting blood glucose; TG, triglycerides; TC, total cholesterol; HDL-c, high-density lipoprotein cholesterol; LDL-c, low-density lipoprotein cholesterol; BUN, blood urea nitrogen; UA, uric acid; CRP, C-reactive protein**

Supplementary TABLE S5. Variance inflation factors (VIF) for covariates in the Cox proportional hazards models

| **Variables** | **GVIF** | **Df** | **GVIF^(1/(2*Df))** |
| --- | --- | --- | --- |
| Gender | 2.403165 | 1 | 1.550215 |
| Marital status | 1.062788 | 1 | 1.030916 |
| Education level | 1.470189 | 2 | 1.101142 |
| Residence | 1.315215 | 1 | 1.146828 |
| Smoking status | 1.953631 | 2 | 1.182254 |
| Drinking status | 1.534924 | 2 | 1.113068 |
| Dyslipidemia | 1.542953 | 1 | 1.242156 |
| Lipid-lowering treatment | 1.51119 | 1 | 1.229304 |
| Hypertension | 2.591975 | 1 | 1.609961 |
| Antihypertensive treatment | 2.52139 | 1 | 1.587888 |
| Diabetes | 2.602065 | 1 | 1.613092 |
| Hypoglycemic treatment | 2.541123 | 1 | 1.59409 |
| Cardiovascular disease | 1.75562 | 1 | 1.324998 |
| Cardioprotective therapy | 1.743135 | 1 | 1.320279 |
| Stroke | 1.553898 | 1 | 1.246554 |
| Cerebroprotective therapy | 1.553224 | 1 | 1.246284 |
| Kidney disease | 1.031489 | 1 | 1.015623 |
| Liver disease | 1.02219 | 1 | 1.011034 |
| Age | 1.532807 | 1 | 1.238066 |
| BMI | 1.012585 | 1 | 1.006273 |
| Waist | 1.233342 | 1 | 1.110559 |
| SBP | 2.58013 | 1 | 1.606278 |
| DBP | 2.33108 | 1 | 1.526787 |
| Nighttime sleep duration | 1.031763 | 1 | 1.015757 |
| HbA1c | 2.089128 | 1 | 1.445382 |
| FBG | 2.0707 | 1 | 1.438993 |
| TG | 5.870955 | 1 | 2.423005 |
| TC | 14.520003 | 1 | 3.810512 |
| HDL-c | 3.113708 | 1 | 1.76457 |
| LDL-c | 12.011197 | 1 | 3.465717 |
| BUN | 1.09779 | 1 | 1.047755 |
| UA | 1.347409 | 1 | 1.16078 |
| CRP | 1.030925 | 1 | 1.015345 |

Abbreviations: BMI, body mass index; SBP, systolic blood pressure; DBP, diastolic blood pressure; HbA1c, glycosylated hemoglobin; FBG, fasting blood glucose; TG, triglycerides; TC, total cholesterol; HDL-c, high-density lipoprotein cholesterol; LDL-c, low-density lipoprotein cholesterol; BUN, blood urea nitrogen; UA, uric acid; CRP, C-reactive protein

Supplementary TABLE S6. Subgroup analyses of the association between the severity of depressive symptoms and risk of incident MCI

| **Subgroup** | **Event/Totals（%）** | **Symptom Severity Stratification HR（95%CI）** | | | **P for interactiom** |
| --- | --- | --- | --- | --- | --- |
|  |  | **No Elevated Symptoms** | **Mild-Moderate Symptoms** | **Severe Symptoms** |  |
| **Age, years** |  |  |  |  | 0.656 |
| ＜60 | 761/5635 (13.5%) | Reference | 1.307(1.111-2.208) [0.001] | 1.557(1.098-2.208) [0.013] |  |
| ≥60 | 515/3826 (13.5%) | Reference | 1.378(1.137-1.670) [0.001] | 1.302(0.855-1.982) [0.220] |  |
| **Gender** |  |  |  |  | 0.364 |
| Male | 628/5203 (12.1%) | Reference | 1.223 (1.0177-1.469) [0.032] | 1.307 (0.852-2.005) [0.220] |  |
| Female | 648/4258 (15.2%) | Reference | 1.433 (1.211-1.695) [0.000] | 1.494 (1.055-2.114) [0.024] |  |
| **Marital status** |  |  |  |  | 0.060 |
| Unmarried | 203/1354 (15.0%) | Reference | 1.129 (0.832-1.533) [0.435] | 1.038 (0.556-1.936) [0.908] |  |
| Married | 1073/8107 (13.2%) | Reference | 1.403 (1.226-1.605) [0.000] | 1.516 (1.125-2.043) [0.006] |  |
| **Education level** |  |  |  |  | 0.010 |
| Elementary school or below | 954/5211 (18.3%) | Reference | 1.230 (1.067-1.417) [0.004] | 1.279 (0.946-1.729) [0.110] |  |
| Middle school or above | 322/4250 (7.6%) | Reference | 1.757 (1.370-2.253) [0.000] | 2.535 (1.412-4.551) [0.002] |  |
| **Residence** |  |  |  |  | 0.384 |
| Urban | 166/2763 (6.0%) | Reference | 1.358 (0.940-1.961) [0.103] | 2.451 (0.955-6.291) [0.062] |  |
| Rural | 1110/6698 (16.6%) | Reference | 1.334 (1.170-1.521) [0.000] | 1.386 (1.048-1.834) [0.022] |  |
| **Smoking status** |  |  |  |  | 0.815 |
| Current | 429/3194 (13.4%) | Reference | 1.300 (1.045-1.618) [0.018] | 1.706 (1.093-2.664) [0.019] |  |
| Former | 105/977 (10.7%) | Reference | 1.494 (0.952-2.343) [0.081] | 1.753 (0.653-4.706) [0.266] |  |
| Never | 742/5290 (14.0%) | Reference | 1.346 (1.147-1.579) [0.000] | 1.254 (0.875-1.798) [0.218] |  |
| **Drinking status** |  |  |  |  | 0.452 |
| Current | 402/3233 (12.4%) | Reference | 1.263 (1.007-1.583) [0.044] | 1.324 (0.773-2.269) [0.307] |  |
| Former | 112/806 (13.9%) | Reference | 1.313 (0.855-2.017) [0.213] | 0.737 (0.258-2.108) [0.570] |  |
| Never | 762/5422 (14.1%) | Reference | 1.393 (1.189-1.631) [0.000] | 1.692 (1.223-2.342) [0.002] |  |
| **Dyslipidemia** |  |  |  |  | 0.824 |
| No | 1161/8374 (13.9%) | Reference | 1.318 (1.158-1.501) [0.000] | 1.389 (1.046-1.845) [0.023] |  |
| Yes | 115/1087 (10.6%) | Reference | 1.626 (1.078-2.454) [0.021] | 1.880 (0.801-4.412) [0.147] |  |
| **Hypertension** |  |  |  |  | 0.782 |
| No | 964/6961 (13.8%) | Reference | 1.357 (1.177-1.564) [0.000] | 1.372 (0.992-1.898) [0.056] |  |
| Yes | 312/2500 (12.5%) | Reference | 1.279 (0.994-1.645) [0.055] | 1.595 (0.982-2.590) [0.059] |  |
| **Diabetes** |  |  |  |  | 0.704 |
| No | 1194/8839 (13.5%) | Reference | 1.324 (1.165-1.505) [0.000] | 1.380 (1.044-1.824) [0.024] |  |
| Yes | 82/622 (13.2%) | Reference | 1.538 (0.932-2.538) [0.092] | 2.420 (0.832-7.033) [0.1.05] |  |
| **Heart disease** |  |  |  |  | 0.505 |
| No | 1138/8242 (13.8%) | Reference | 1.367 (1.200-1.558) [0.000] | 1.522 (1.140-2.033) [0.004] |  |
| Yes | 138/1219 (11.3%) | Reference | 1.055 (0.725-1.535) [0.780] | 0.922 (0.450-1.889) [0.824] |  |
| **Stroke** |  |  |  |  | 0.827 |
| No | 1250/9252 (13.5%) | Reference | 1.339 (1.182-1.517) [0.000] | 1.446 (1.100-1.900) [0.008] |  |
| Yes | 26/209 (12.4%) | Reference | 1.284 (0.436-3.782) [0.650] | 1.965 (0.369-10.476) [0.429] |  |

Note: Table displays hazard ratios (squares) and 95% confidence intervals (horizontal lines) for the association between depressive symptoms (vs. no symptoms) and MCI risk across various subgroups. The size of the square represents the sample size of the subgroup. The model was fully adjusted (Model 5). P for interaction was calculated.

Supplementary TABLE S7. **Comparison of the associations between depressive symptoms and risk of MCI in the primary and sensitivity analyses**

| **Model and Analytical Approach** | **Exposure Group (vs. No Elevated Symptoms)** | **95%CI** | **P-value** |
| --- | --- | --- | --- |
| Primary Analysis |  |  |  |
|  | Mild-Moderate Symptoms | 1.363 (1.205-1.542) | <0.05 |
|  | Severe Symptoms | 1.468 (1.123-1.918) | <0.05 |
| Sensitivity Analysis 1 |  |  |  |
|  | Mild-Moderate Symptoms | 1.201 (1.062-1.358) | <0.05 |
|  | Severe Symptoms | 1.167 (0.891 - 1.528) | 0.261 |
| Sensitivity Analysis 2 |  |  |  |
|  | Mild-Moderate Symptoms | 1.306 (1.139-1.498) [OR] | <0.001 |
|  | Severe Symptoms | 1.343 (0.992-1.819) [OR] | 0.056 |

Note: This table compares the hazard ratios (HR) or odds ratios (OR) and their 95% confidence intervals (CI) and P-values for the association between varying severity of depressive symptoms and the risk of MCI from the primary analysis and two sensitivity analyses. Primary Analysis: A Cox proportional hazards regression model was used to assess the prospective association between depressive symptoms and the risk of incident MCI. Sensitivity Analysis 1: To address potential bias from temporal variations in assessment tools or diagnostic criteria, a stratified Cox model was fitted using assessment year as a stratification variable, allowing for separate baseline hazards for each period. Sensitivity Analysis 2: To account for the competing risk of death and utilize all intermittent follow-up data, a multinomial logistic regression model was implemented with a composite outcome (MCI-free, incident MCI, all-cause death). The odds ratios (OR) presented here represent the association of depressive symptoms with incident MCI, using the MCI-free group as the reference.

Supplementary FIGURE S1. Data source

**
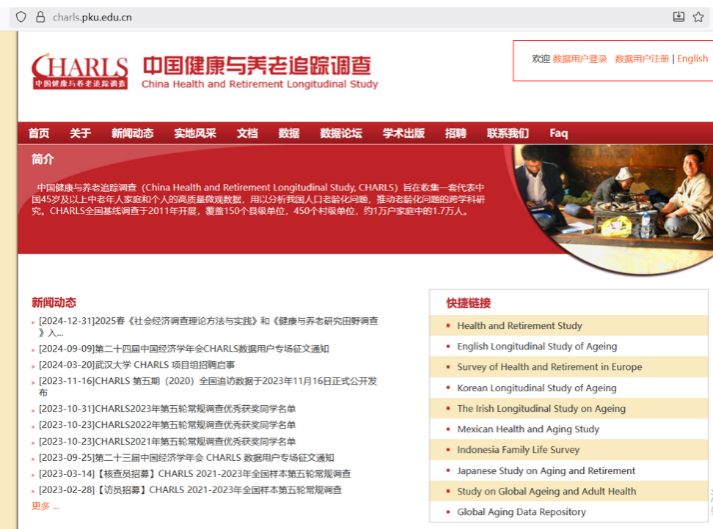
**

Supplementary FIGURE S2. Data access


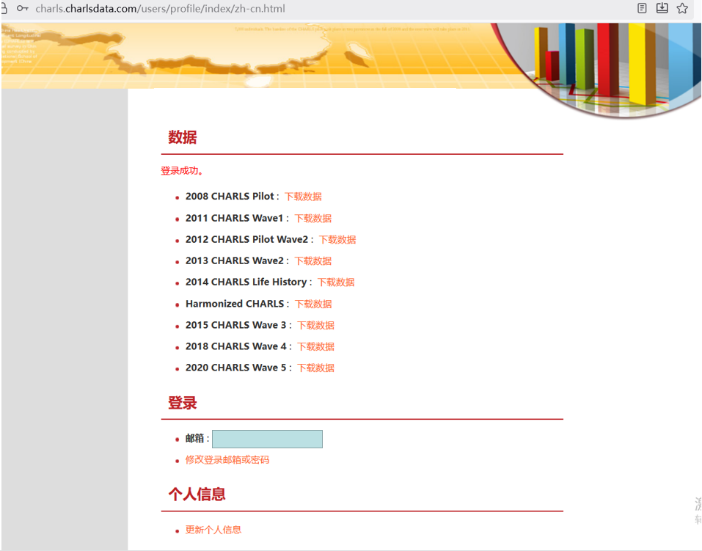


Supplementary TABLE S8. Data analysis flowchart

| **Analysis Phase** | **Primary Tool** | | **Core Tasks** |
| --- | --- | --- | --- |
| Data Preprocessing | R | Primarily using dplyr, tidyr packages | Data cleaning, variable merging and transformation, missing value imputation, data format conversion, etc. |
| Core Statistical Analysis | SPSS |  | Descriptive statistics, hypothesis testing (t-tests, chi-square tests, etc.), Cox or logistic regression, etc.) |
| Restricted Cubic Spline Analysis | R | Primarily using the rms package | Fitting restricted cubic spline models to explore the non-linear relationship between continuous independent variables and the outcome. |
| Visualization | GraphPad Prism |  | Generating publication-ready, high-quality forest plots based on the statistical analysis results. |
